# Development of tools for quantitative intracellular metabolomics of *Aspergillus niger* chemostat cultures

**DOI:** 10.1007/s11306-015-0781-z

**Published:** 2015-02-25

**Authors:** Francisca Lameiras, Joseph J. Heijnen, Walter M. van Gulik

**Affiliations:** Cell Systems Engineering Section, Department of Biotechnology, Delft University of Technology, 2628 BC Delft, The Netherlands

**Keywords:** *Aspergillus niger*, Chemostat, Rapid sampling, Cold methanol quenching, Quantitative metabolomics

## Abstract

In view of the high citric acid production capacity of *Aspergillus niger*, it should be well suited as a cell factory for the production of other relevant acids as succinic, fumaric, itaconic and malic. Quantitative metabolomics is an important *omics* tool in a synthetic biology approach to develop *A. niger* for the production of these acids. Such studies require well defined and tightly controlled cultivation conditions and proper rapid sampling, sample processing and analysis methods. In this study we present the development of a chemostat for homogeneous steady state cultivation of *A. niger*, equipped with a new dedicated rapid sampling device. A quenching method for quantitative metabolomics in *A. niger* based on cold methanol was evaluated using balances and optimized with the aim of avoiding metabolite leakage during sample processing. The optimization was based on measurements of the intermediates of the glycolysis, TCA and PPP pathways and amino acids, using a balance approach. Leakage was found to be absent at −20 °C for a 40 % (v/v) methanol concentration in water. Under these conditions the average metabolite recovery was close to 100 %. When comparing *A. niger* and *Penicillium chrysogenum* metabolomes, under the same cultivation conditions, similar metabolite fingerprints were found in both fungi, except for the intracellular citrate level which is higher for *A. niger*.

**Electronic supplementary material:**

The online version of this article (doi:10.1007/s11306-015-0781-z) contains supplementary material, which is available to authorized users.

## Introduction

Filamentous fungi like *Aspergillus niger*, form a key step in the global carbon cycle by their capacity to degrade plant cell wall wastes efficiently, as they have one essential advantage: the massive secretion of enzymes that are capable of degrading plant cell wall constituents into available sugars which can subsequently be taken up by the cells. This fungal characteristic is already exploited industrially in the production of enzymes such as glucoamylases and hemicellulases. Moreover, *A. niger* is applied in large scale industrial fermentations for the production of citric acid.

Considering the high citric acid production capacity of *A. niger* at low pH, it should be well suited for the production of other relevant organic acids, such as itaconic, succinic, fumaric and malic acid. Bio-based fermentative production of these acids from plant waste streams using *A. niger*, is an attractive alternative to their petroleum-based production. Moreover, the mentioned dicarboxylic acids have a good potential as chemical building blocks in polymer synthesis (Werpy et al. 2004). Overproduction of dicarboxylic acids in *A. niger* requires metabolic engineering of the fungus, not only with respect to the product pathway but also with respect to the import of lignocellulosic sugars (glucose, xylose, arabinose, etc.) and the export of the produced acids.

*A. niger* as a cell factory exhibits a flexible metabolism which enables growth on a wide range of substrates. Furthermore, its genome has been fully sequenced (Pel et al. 2007) which facilitates metabolic engineering efforts for the development of strains for the production of new compounds and subsequent strain and process improvement. Hence, a systems biology approach can be applied for identifying and removing bottlenecks by combining different—*omics* levels.

Steady state as well as dynamic quantitative metabolomics with and without stable isotope labelling can be applied to identify kinetic and capacity bottlenecks in the product pathway, substrate import and product export. Such metabolomics studies require well defined and tightly controlled cultivation conditions and proper rapid sampling, sample processing and analysis methods (van Gulik et al. 2000).

Unfortunately, the filamentous growth-form of *A. niger* poses problems, especially in bench-scale fermentors, due to the tendency of the organism to grow as pellets and to accumulate on the walls and probes of the fermentor, as well as in the outflow system in case of chemostat cultivation (Schrickx et al. 1993; Larsen et al. 2004). This should be avoided as a homogeneous culture is a prerequisite for proper metabolomics/fluxomic studies. Due to these practical difficulties, little work has been done in the direction of chemostat cultivation of *A. niger*.

In one of the few chemostat studies on *A. niger*, changes in mycelium morphology and conidia formation were studied as a function of the growth rate (Schrickx et al. 1993). In the same work a Teflon covered ring bar magnet was used, which could be moved over the wall with an external horseshoe magnet, to remove wall growth. With a similar purpose, Swift et al. 1998 tried to reduce biomass accumulation on internal surfaces by periodically increasing the stirrer speed for 5–10 min.

Larsen et al. (2004) suggested the use of a custom made *variomixing* bioreactor, in which intermittently rotating baffles reduce the surface area susceptible to wall growth and probes were inserted below the surface level of the culture to prevent mycelium accumulation between the probes. With this technically complex bioreactor, wall growth was significantly reduced in batch cultivations of *Aspergillus oryzae*. Later on this reactor was successfully used for steady state chemostat cultivation of *A. niger* (Jørgensen et al. 2007). With the purpose of minimization of wall growth, Jørgensen et al. 2011 cooled the glass surface of the headspace of the bioreactor. Another problem when growing *A. niger* is its aggregation as pellets. It has been reported that when inoculating a culture with spores at pH values of 3.5 or higher, pellets were formed whereas free mycelium was formed when inoculation was done at pH 2.5 (Pedersen et al. 2000).

In addition to a homogeneous cultivation, accurate sampling is required for quantitative metabolome analysis. Over the years, different rapid sampling devices have been developed (Schädel and Franco-Lara 2009), to allow fast sampling of biomass from bioreactors for intracellular metabolomics studies. When constructing a sampling device, the residence time for the cells to pass from the reactor to a quenching fluid has to be considered and compared to the consumption rate of the available substrate and oxygen. This residence time should be short enough to prevent any change in limitation to occur and thus to prevent changes in metabolite levels during sampling. Additionally, dead zones within the equipment must be avoided and the construction must permit aseptic use (Larsson and Törnkvist 1996). In most of the described sampling devices the dead volume has been reduced by using channels with a small internal volume such as HPLC capillaries.

Currently, there is a sampling device built in house (Lange et al. 2001), designed for *Saccharomyces cerevisiae.* This sampling device has been used for other metabolomics studies in different organisms, as *Escherichia coli,* *Pichia pastoris* and even the filamentous *Penicillium chrysogenum* (Carnicer et al. 2012; Taymaz-Nikerel et al. 2009; de Jonge et al. 2012). However, *A. niger*, being a filamentous fungi, was not suited for the usage of such a device comprehending a sampling tube of 0,8 mm, and thus blocking the system due to long hyphae (100 µm) of the organism (Swift et al. 1998).

Fast changes in the environment of the cells directly influences their metabolism and thus also the outcome of a metabolome analysis. It is known that the turnover times of intracellular metabolites are generally small, in the order of seconds, considering their conversion rates and intracellular concentrations. Hence, quenching of cellular metabolism within a fraction of a second upon sampling is required, in order to avoid further (inter)conversion of metabolites and obtain a proper snapshot of the metabolic state.

The most critical assumption in quenching methods which allow separation of cells and extracellular medium and washing of the biomass, such as the cold methanol/water method (de Koning and van Dam 1992) is that intracellular metabolites will remain inside the cells during quenching, separation and washing. However it is known that some metabolites can leak from the cells into the quenching solution, which is later discarded, thus underestimating intracellular levels (Canelas et al. 2008). For eukaryotic microorganisms, leakage can be avoided by adaptation of the composition of the quenching fluid (Carnicer et al. 2012; de Jonge et al. 2012; Canelas et al. 2008). Apart from quenching, also a proper extraction procedure is crucial, as it is not desired that the metabolites of interest are (inter)converted and/or degraded during this procedure. Only very few studies have been performed on the optimization of quenching and extraction procedures for metabolomics of *A. niger*.

Ruijter and Visser (1996) performed rapid sampling of *A. niger* in a quenching solution of 60 % aqueous methanol at −45 °C and metabolite extraction was performed using a cold chloroform/methanol method. In this study it was stated that metabolites do not leak from the cells, as no significant concentrations were detected in the quenched filtrate. However the detection limits of the analysis were not specified, and leakage cannot be quantified from intracellular concentrations only, as one needs to compare external and intracellular metabolite amounts. Jernejc (2004) evaluated different quenching methods and different extraction methods for metabolite recovery in *A. niger*. Quenching in liquid nitrogen or in a 60 % methanol water solution at −40 °C, gave similar results. For metabolite extraction, acid and alkali extractions were considered better methods than ethanol extraction, though recoveries of the different methods were not checked. In these previous protocols validation of absence of leakage was not performed.

Here we describe the successful development of a chemostat protocol for homogeneous steady state cultivation of *A. niger,* based on a conventional turbine stirred bioreactor. We equipped the reactor with a dedicated rapid sampling device for fast and reproducible withdrawal of mycelium samples, allowing quantitative metabolome analysis. To obtain reliable snapshots of the metabolome, a cold methanol/water based quenching procedure was optimized and validated for absence of leakage for *A. niger* using the metabolite balance approach described by Canelas et al. (2008). To this end we quantified an extensive set of metabolites with different physicochemical properties in different sample fractions, using isotope dilution mass spectrometry (Mashego et al. 2004; Wu et al. 2005).

## Materials and methods

### Strain and inoculum

The strain used was *A. niger* NW185. The conidial inocula for chemostat cultivation were obtained from cultures on complete medium agar plates at pH 6 containing 9 g/L glucose monohydrate as carbon source, 6 g/L NaNO_3_ as N-source, 1.5 g/L KH_2_PO_4_, 0.5 g/L KCl, 0.5 g/L MgSO_4_·7H2O, 2 g/L meat peptone, 1 g/L yeast extract, 1 g/L tryptone and 15 g/L agar.

The agar medium was supplemented with 1 mL/L of trace elements solution, containing 10 g/L EDTA, 4.4 g/L ZnSO_4_·7H_2_O, 1.0 g/L MnCl_2_·4H_2_O, 0.32 g/L CoCl_2_·6H_2_O, 0.32 g/L CuSO_4_·5H_2_O, 0.22 g/L (NH_4_)_6_Mo_7_O_24_·4H_2_O, 1.47 g/L CaCl_2_·2H_2_O and 1.0 g/L FeSO_4_·7H_2_O (Vishniac and Santer 1957) and 1 mL/L of vitamins solution (containing 0.05 g/L D-biotin, 1 g/L CaD(+)panthotenate, 1 g/L nicotinic acid, 25 g/L myo-inositol, 1 g/L thiamine chloride hydrochloride, 1 g/L pyridoxol hydrochloride, 0.2 g/L p-aminobenzoic acid).

Media were sterilized at 121 °C for 20 min and the glucose solution was sterilized separately at 110 °C. The trace elements and vitamin solutions were added sterile to the culture media by filtration through a 0.2 µm cartridge filter (Whatman FP 30/0.2 CA-S).

One week before the inoculation of the fermentor cultures, the medium plates were inoculated with conidia from a stock culture kept at 4 °C. Plates were incubated at 30 °C for 4 days and then stored at 4 °C. Spores were harvested and washed with saline solution containing 0.9 % NaCl and 0.05 % Tween 80 to enhance the release of the spores from the plates.

### Fermentor set up

A 7 L turbine-stirred bioreactor was used (Applikon, Delft, The Netherlands). The bioreactor was set up and operated as described in Nasution et al. (2006) with the following differences:

The broth level was chosen such that the baffles were completely covered (working volume of 4.5 ± 0.01 L). A cooling tubing (4 °C) was wrapped around the headspace of the fermentor to minimize growth of the fungus on the glass surface above the liquid. The exhaust gas of the fermentor was passed through a condenser (4 °C) and a Nafion dryer (Permapure, Toms River, USA) before entering a NGA 2000 offgas analyser (Rosemount Analytical, Anaheim, USA) for measurement of the oxygen and carbon dioxide contents. Data acquisition was performed with MFCS/win 3.0 software.

The bioreactor was equipped with pH, temperature and dissolved oxygen probes, inserted in the metal bottom part of the fermentor, below the broth level.

The cultivation temperature was kept constant at 30.0 °C (±0.1), and the pH (Mettler Toledo InPro^®^ 3030/120) was controlled at 3.0 (±0.1) by addition of 4 M H_2_SO_4_.

Sterile air was supplied via a mass flow controller (Brooks 58505 calibration at 0 °C and 1 bar) at 1 L/min through a bottom sparger. Dissolved oxygen was monitored with an AppliSens Z010011020 probe.

To avoid biofilm formation on the wall, continuous media inflow was supplied at the bottom of the fermentor, along with the air supply. An overpressure of 0.3 bar was applied.

### Batch cultivation

The fermentor, containing 4.5 kg of minimal medium, was inoculated with a conidial suspension to give a final concentration of 1 × 10^6^ spores/mL, along with 0.003 % (wt/wt) of yeast extract to induce conidia germination (Jørgensen et al. 2011).

The glucose limited minimal medium contained 0.5 g/L (NH_4_)_2_SO_4_, 0.3 g/L KH_2_PO_4_, 3 g/L NH_4_H_2_PO_4_, 0.5 g/L MgSO_4_·7H_2_O and 1 mL/L trace elements solution (composition as described above), with a final pH of 4.6. Sterilization of the medium was performed by passing it through a 0.2 µm filter (Sartorius stedim Sartopore2). The substrate concentration was 7.5 g/L glucose monohydrate and the fermentor pH was controlled at 2.5 to prevent pellet formation.

Oxygen-sufficient growth was ensured by preventing the dissolved oxygen tension (DO) to decrease below 50 %, of air saturation by automatic DO control through progressively increasing the stirrer speed from initial 100 rpm to a maximum of 450 rpm at the end of batch fermentation.

### Chemostat cultivation

Continuous cultivation was started in the late exponential phase of the batch culture, approximately 30 h after inoculation, by starting the medium feed. The composition of the chemostat medium was the same as the batch medium. Effluent was removed discontinuously via a pneumatic valve in the bottom of the reactor and a peristaltic effluent pump controlled by the broth weight. During chemostat cultivation the stirrer speed was kept constant at 450 rpm.

### Sampling and analytical procedures

When a steady state was reached (after five residence times), at least six samples were taken for quantification of cell dry weight, concentrations of extracellular compounds and TOC (total organic carbon) content.

Broth samples for determination of the biomass dry weight concentration were taken from three different sections of the fermentation setup: the regular sampling port aside the fermentor vessel, the sampling port of the rapid sampling device and the outflow tube upstream the effluent vessel. In all cases, biomass dry weight was determined by filtration over glass fibre filters (47 mm, type A/E, Pall, USA), subsequent washing with water and drying at 70 °C until constant weight. All samples were analysed in triplicate.

Filtrate samples were obtained by quickly (within 3 s) withdrawing 5 mL of broth, via the over-pressure on the fermentor, into a syringe containing cooled steel beads (−20 °C) to bring the sample temperature down to 0 °C (Mashego et al. 2003). The broth was then immediately pressed through a 0.45 µm cartridge filter (Millex-HV durapore PVDF membrane) into a sampling tube, which was directly frozen in liquid nitrogen.

The total organic carbon content of the filtrate samples was quantified with a total organic carbon analyser (type TOC-L, Shimadzu, Kyoto, Japan). With this method, both total carbon (TC) and inorganic carbon (TIC) were measured. The latter representing the content of dissolved carbon dioxide and carbonic acid salts. Subtracting the inorganic carbon from the total carbon, yields the TOC.

The morphology of the hyphae was inspected by microscopy (Axiostarplus, Carl Zeiss, Jena, Germany) using a 100× objective, and biomass elemental composition was determined by combustion and subsequent gas analysis (carbon dioxide, water vapour and nitrogen mass fractions), gas chromatography (oxygen) and ICP-MS (phosphorus and sulphur) (Energy Research Centre of the Netherlands).

### Rapid sampling and quenching

**Fig. 1:**
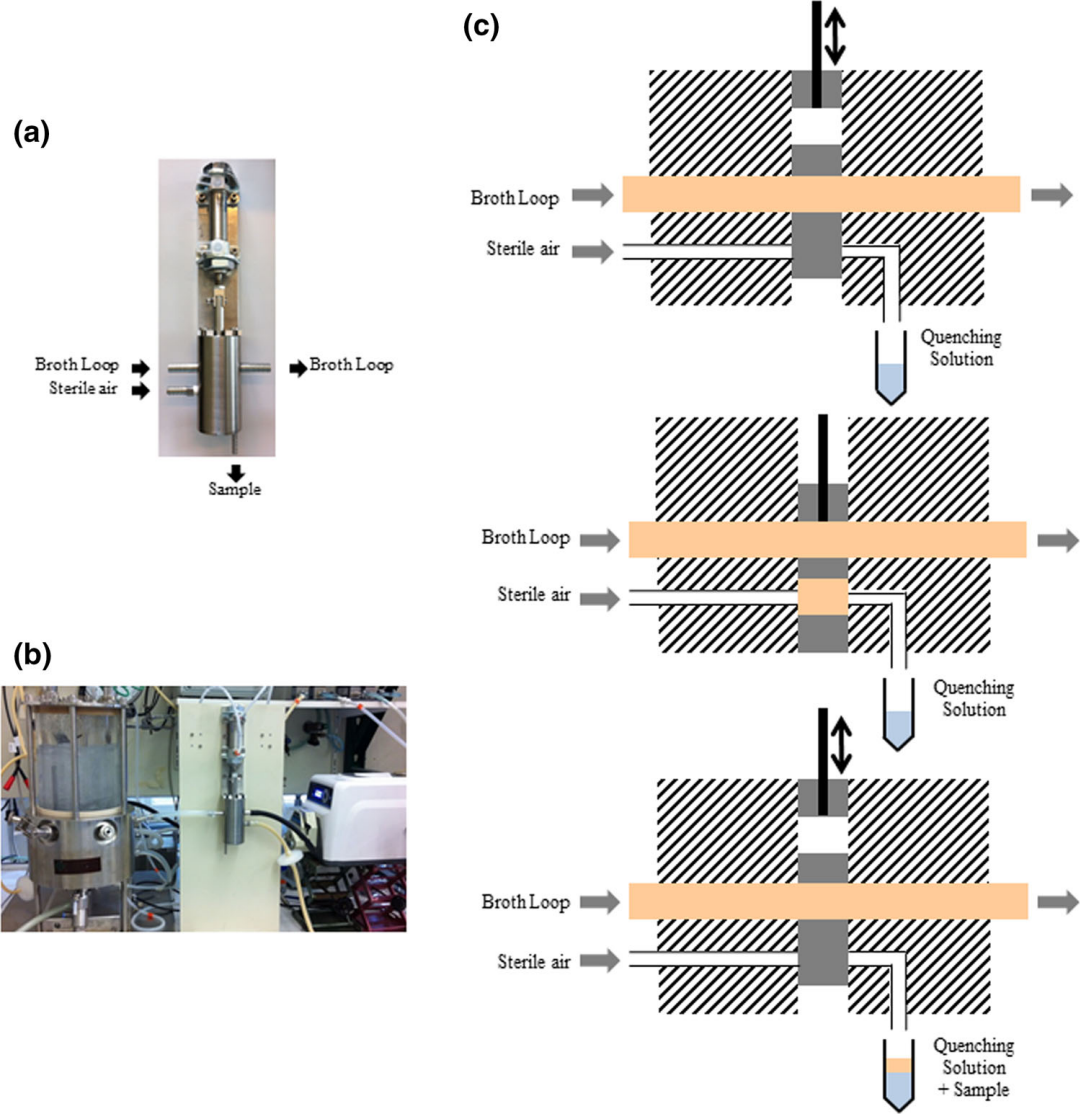
Custom-made rapid sampling device and its working principle

A new customized rapid sampling device (Fig. 1a, b) designed such that pellet formation would not result in blockage of the device was built in house. The working principle is shown in Fig. 1c.

Via a loop with an internal diameter of 8 mm and a peristaltic pump (Masterflex L/S, Cole-Parmer), the broth was pumped from the fermentor through the sampling device and back into the fermentor at a flow rate of 40 mL/s. The broth residence time in the part of the loop between the fermentor broth outflow and the sampling port was 0.25 s. The broth residence time in the entire loop was 1.3 s. If metabolite dynamics has to be performed, the highest frequency of this device is 3 s, at which samples can be sequentially taken.

When sampling, the piston of the sampling device is pushed down and up again by a two-way pneumatic cylinder, removing a constant volume of broth from the loop. This constant volume is then pushed into a sampling vial containing the cold quenching fluid, by a constant flow of sterile air.

#### Rapid sampling and quenching for intracellular metabolome quantification

**Fig. 2:**
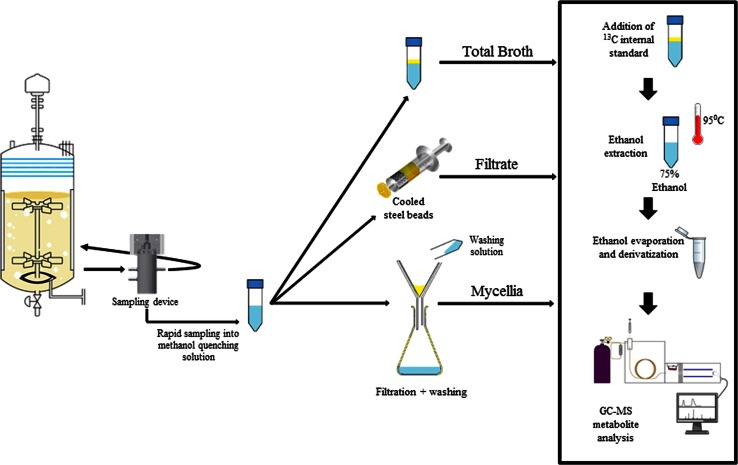
Rapid sampling and quenching procedure of *A. niger*

The procedure is described for the standard 60 % aqueous/methanol quenching solution, however, the same procedure was applied for 50 % (at −20 °C) and 40 % (at −20 °C) aqueous/methanol mixtures (Fig. 2).

Two times 0.76 (±0.04) g of broth was rapidly withdrawn from the broth loop with the sampling device and injected (<1 s) into the same vial containing 10 mL of 60 % aqueous methanol (v/v) solution pre-cooled to.

−40 °C. The exact sample weight was determined by weighing each tube before and after sampling. The content of each tube was quickly mixed by vortexing (1 s) and the tube was placed back in a cryostat (Lauda, Germany) at −40 °C. A set of three replicates were taken within 1 min.

The quenched cold mycelia samples were harvested and washed by cold filtration, according to a previously described method (Douma et al. 2010) which was slightly modified. Filtration was performed using glass fibre filter disks (PALL glass fibre type E). To cool down the filter and filter unit 20 mL of 60 % aqueous methanol (v/v) solution pre-cooled to -40 °C was poured onto the filter. Immediately thereafter the quenched mycelium sample was poured into the cold aqueous methanol on the filter. Then the vacuum pump was turned on and after the filter fell dry, 20 mL of cold (−40 C) 60 % methanol was poured on the filter for a first washing followed by 40 mL of cold 60 % methanol for a second washing of the mycelium sample.

#### Rapid sampling and quenching for extracellular metabolite quantification

Two times 0.76 (±0.04) g of broth, withdrawn with the rapid sampling device, was quickly filtered through a syringe containing cooled steel beads (−20 °C) and a 0.45 µm cartridge filter (Millex-HV durapore PVDF membrane) directly into the same tube containing 10 ml 40 % methanol (v/v) at −20 °C. The exact sample weights were determined by weighing all tubes before and after sampling.

#### Rapid sampling and quenching for metabolite quantification in whole broth

Sampling was done as for intracellular metabolites, by quenching two times 0.76 (±0.04) g of broth in 10 mL of 40 % aqueous methanol (v/v) solution pre-cooled to −20 °C, but the quenched cell suspension was not filtered. The exact sample weights were determined by weighing all tubes before and after sampling and transfer.

#### Extraction method

Boiling ethanol extraction was performed to ensure complete cell disruption and inactivation of enzyme activity, according to a method modified from Lange et al. 2001. The three sample fractions (described in 2.6.1–2.6.3) were extracted immediately after sampling and quenching. For intracellular samples, the cooled filter paper with quenched mycelium was placed in a falcon tube containing 25 mL of 75 % ethanol (pre-heated at 75 °C), along with 100 µL of U-^13^C-labeled cell extract of *S. cerevisiae* as internal standard. Each tube was immediately vortexed and placed in a water bath at 95 °C. After 3 min each tube was placed on ice, and later stored at −80 °C. Extracellular and total broth fractions were also submitted to the same process, by adding the whole volume of quenched solution into 25 mL of 75 % ethanol, with 100 µL of U-^13^C-labeled yeast extract, and boiled for 3 min at 95 °C, as described for intracellular fractions.

#### Ethanol evaporation

All extracts, were evaporated to dryness in a Rapid-Vap (Labconco, Kansas City, MO) under vacuum for 240 min, based on the protocol from Mashego et al. 2004. After evaporation, the residues were resuspended in 500 µL of water, and centrifuged at 1,000 g to remove cell debris. The supernatants were stored at −80 °C until further analysis.

#### Metabolite quantification

The concentrations of the metabolic intermediates from glycolytic, TCA and PPP pathways and aminoacids were measured by isotope dilution mass spectrometry (LC-IDMS/MS and GC-IDMS) according to the protocols of van Dam et al. 2002, de Jonge et al. 2011and Cipollina et al. 2009 (Table S1).

### Balances, biomass specific rates and data reconciliation

From compound balances, the rates of substrate (R_S_), biomass (R_X_), O_2_ ${\text{R}}_{{{\text{O}}_{2} }}$ and by-products (R_TOC_) were calculated. From these rates, carbon and degree of reduction recoveries (Eqs. 1 and 2) were calculated wherein possible excretion of by-products was quantified by measurements of the total organic carbon content of the culture filtrate.

1 $${\text{Carbon}}\;{\text{recovery}}\; ( {{\% )}} = \frac{{\text{R}}_{\text{biomass}} \; + \;{\text{R}}_{{{\text{CO}}_{ 2} }} \; + \;{\text{R}}_{\text{toc}}}{{\text{R}}_{\text{substrate}}} \;\times 100$$

2 $${\text{Degree}}\,\text{{of}}\,{\text{reduction}}\left( {{\% }} \right){ = }\frac{{\gamma_{\text{biomass}} {\text{R}}_{\text{biomass}} + \gamma_{\text{toc}} {\text{R}}_{\text{toc}} + \gamma_{{{\text{O}}_{ 2} }} {\text{R}}_{{{\text{O}}_{ 2} }} }}{{\gamma_{\text{substrate}} {\text{R}}_{\text{substrate}} }} \times 100$$

The biomass specific net conversion rates, i.e. growth rate, glucose and oxygen consumption rates and carbon dioxide and TOC production rates, were calculated from the primary measurements of concentrations in gas and liquid phases, as well as gas and liquid flow rates, using the corresponding steady state mole balances. Data reconciliation was applied to obtain the best estimates of the measurements, within their error margins, using the elemental and charge conservation relations as constraints (Verheijen 2010).

### Metabolite consistency check

The extent of metabolite leakage was evaluated by a metabolite balance approach according to Canelas et al. 2008. Each metabolite was quantified in different sample fractions, that is, in the mycelium fraction (intracellular; IC), the culture filtrate (extracellular; EC) and in the whole broth (WB). As the ^13^C internal standard mix was added upstream the extraction step, possible metabolite losses due to partial degradation were corrected for.

For each metabolite i, the following metabolite balances should be satisfied, if leakage of i from the cells into the quenching solution is absent:

3 $$\text{M}_{i} (TB) = M_{i} (IC) + M_{i} (EC)$$

Here, Mi is the total amount of metabolite present per gram biomass dry weight.

If leakage occurs, the amount released from cells into the quenching solution can be calculated from:

4 $$\text{M}_{i} (leakage) = M_{i} (TB) - M_{i} (IC) - M_{i} (EC)$$

Finally, the metabolite recovery can be calculated as:

5 $${\text{Recovery}}\; ( {\text{\% )}} = \frac{{{\text{M}}_{\text{i}} ( {\text{IC)}}}}{{{\text{M}}_{\text{i}} ( {\text{TB) - M}}_{\text{i}} ( {\text{EC)}}}} \times 100$$

## Results and discussion

### Chemostat cultivation of *A. niger*

#### Development of the chemostat protocol

*Aspergillus niger* was grown in a 7 L turbine stirred bioreactor, specifically constructed for chemostat cultivation of filamentous fungi. The reactor vessel consisted of a glass upper part and a stainless steel bottom part in which all probes were inserted. The stirrer was driven from the bottom, by means of a magnetic coupling. Feed medium was supplied continuously from the top of the reactor while effluent removal was discontinuous, accomplished with a pneumatic outflow device and a peristaltic pump which were controlled by broth weight.

Despite these features, a homogeneous steady state chemostat culture could not be achieved in this reactor, due to massive wall growth and accumulation of biomass in the headspace of the reactor (Fig. S1), as also experienced in other studies (Schrickx et al. 1993; Larsen et al. 2004).

In order to obtain a homogeneous cultivation, avoiding accumulation of mycelium above the liquid surface and on the glass wall, as well as avoiding pellet formation, both the chemostat system and the way of operation had to be adapted.

As massive mycelium growth occurred in the headspace of the reactor below the medium inlet, which was positioned in the lid of the vessel, the medium inflow was moved to the sterile air supply, at the bottom of the fermentor. The broth level was chosen such that it covered the baffles to prevent biomass adhesion (4.5 L working volume) and a cooling coil (4 °C) was wrapped around the headspace of the fermentor with the same purpose. Moreover, a low cultivation pH (2.5) was used to prevent pellet formation.

During the preceding batch phase, the stirrer speed was progressively increased from 100 rpm to a maximum of 450 rpm through DO control (setpoint 50 %), which minimized splashing and consequently biofilm formation on the fermentor wall. With these adaptations, homogeneous steady state chemostat cultivations could be achieved without significant wall growth. An illustrative figure can be found in the supplementary material Fig. S2.

Effluent broth was discontinuously removed via an 8 mm diameter tubing to assure homogeneous withdrawal of broth, where the biomass concentration in the broth outflow and in the fermentor are the same. To verify this, in a previous chemostat study at dilution rates of 0.045 and 0.087 h^−1^ (results not shown), the biomass concentration was measured in the outflow (C_x,out_) and compared with the biomass concentration (C_x_) inside the fermentor (Table S2), showing no significant difference. Consequently, the important condition for a proper chemostat cultivation, that is, C_x_ = C_x,out_, was satisfied.

#### Batch and chemostat cultivations

Using the optimized fermentor set up, *A. niger* was grown in aerobic glucose limited chemostat culture. During the preceding batch phase, the pH was controlled at 2.5, which avoided the formation of pellets and assured exponential growth. The batch maximum growth rate on glucose was 0.26 (±0.01) h^−1^. This value is somewhat lower than the value of 0.29 h^−1^ reported in Jørgensen et al. 2007, for a different strain of *A. niger*.

During the late exponential phase, chemostat cultivation was initiated. Four different dilution rates were applied. Cultivations were assumed to be in steady state after five residence times under glucose limitation, which was verified by the measured carbon dioxide (Fig. S3) and oxygen concentrations in the offgas being constant in time.

For all dilution rates, the biomass concentration was determined by dry weight, and the mycelium was examined microscopically, revealing no differences in morphology (Table 1), showing polarized growth of each hyphal tip and an increasing number of branches, resulting in freely dispersed mycelia in all cases.

**Table 1:** Applied chemostat effluent flow rates and resulting dilution rates and obtained steady state biomass concentrations and respective biomass morphology at different dilution rates

Dilution rate (h^−1^)	0.043 ± 0.001	0.089 ± 0.001	0.134 ± 0.001	0.205 ± 0.003
Effluent flow rate (mL/h)	193	402	599	922
Residence time (h)	23.30	11.18	7.49	4.87
Biomass concentration (g_DW_/Kg_broth_)	3.02 ± 0.03	3.25 ± 0.04	3.38 ± 0.03	3.45 ± 0.05
Morphology (100× amplification)				

#### Steady state and data reconciliation

For all dilution rates, measurements of residual glucose, biomass concentration, oxygen and carbon dioxide concentrations in the offgas were performed. In addition the total organic carbon (TOC) concentration of the culture filtrate was determined in order to verify whether significant amounts of by-products were formed. From these measurements, it appeared that recoveries of the carbon and degree of reduction were >90 % and thus the biomass specific rates were calculated and reconciled (Table 2). Moreover, the biomass elemental composition was determined from several chemostat samples and shown to be 27.3 ± 0.2 g/mol (C_1_H_1.8_N_0.12_O_0.6_) (Table S3).

**Table 2:** Biomass specific net conversion rates and carbon and redox recoveries of steady state chemostat cultivations carried out at different dilution rates

Dilution rate (h^−1^)	0.043		0.089		0.134		0.205	
	Unreconciled	Reconciled	Unreconciled	Reconciled	Unreconciled	Reconciled	Unreconciled	Reconciled
Carbon recovery (%)	101 ± 11	–	97 ± 13	–	98 ± 12	–	95 ± 12	–
Redox recovery (%)	99 ± 6	–	97 ± 4	–	95 ± 3	–	92 ± 3	–
q_x_ (mCmol/h)/Cmol)	42.9 ± 12.4	42.9 ± 3.5	89.45 ± 28.3	89.5 ± 5.2	133.5 ± 39.4	133.5 ± 5.0	205.3 ± 59.3	205.3 ± 6.7
q_S_ (mmol/h)/Cmol	14.0 ± 2.9	14.2 ± 0.8	27.1 ± 6.1	25.5 ± 1.1	38.8 ± 8.1	37.23 ± 1.0	58.5 ± 11.9	52.96 ± 1.2
${\text{q}}_{{{\text{o}}_{2} }}$ (mmol/h)/Cmol	33.1 ± 20.0	35.3 ± 2.2	54.4 ± 20.9	52.8 ± 2.5	78.0 ± 22.0	78.7 ± 2.8	106.5 ± 26.7	96.4 ± 3.2
${\text{q}}_{{{\text{co}}_{2} }}$ (mmol/h)/Cmol	35.1 ± 11.6	36.6 ± 2.3	64.7 ± 25.0	55.5 ± 2.6	91.9 ± 33.9	82.6 ± 2.9	134.6 ± 50.3	102.4 ± 3.3
q_TOC_ (mCmol/h)/Cmol)	5.9 ± 2.7	5.9 ± 2.4	8.4 ± 4.2	8.1 ± 3.5	7.5 ± 3.5	7.3 ± 2.9	10.9 ± 5.0	10.1 ± 4.0

In further analysis, the *Herbert*-*Pirt* equation was obtained for this typical example of aerobic growth, whereby the yield and the maintenance coefficients were estimated (Fig. S4) with respective values of 0.270 ± 0.002 mol_S_/Cmol_X_ and 2.6 × 10^−3^ ± 0.3 × 10^−3^ mol_S_/h.Cmol_X_ (=0.1 mmol_S_/h.g_DW_). These values are of the same order of magnitude with another glucose limited chemostat study, where Y_X/S_ and m_S_ were reported as 2.14 µmol/g_DW_ (≈0.2 mol_S_/Cmol_X_) and 0.16 µmol/h.g_DW_ (=0.5 mmol_S_/h.g_DW_) respectively (Jørgensen et al. 2007). In another study (Roels 1983), the maintenance coefficient for several microorganisms was also shown to be in the same order of magnitude as presented here.

Growth conditions were glucose limited (residual glucose concentration around 0.6 µCmol/L at 0.043 h^1^) and aerobic, however TOC measurements revealed that the culture filtrates contained a significant amount of organic carbon, corresponding to 2–7 % of the carbon supplied as glucose (15.8 mCmol/L at 0.043 h^−1^). Higher TOC percentages were found for lower dilution rates (Fig. S5). As the concentrations of residual glucose were considerably lower, the measured residual carbon was credited to cell lysis and excreted enzymes, since no significant amounts of organic acids were detected extracellularly (results not shown).

### Rapid sampling and quenching

#### Performance of the rapid sampling device

A proper sampling procedure is essential for unbiased quantification of the intracellular metabolome. Therefore, a sampling device that enables fast withdrawal of homogeneous broth samples which can in principle be operated at high sampling rates on a sub second scale was constructed (see materials and methods). High sampling rates are desired for future experiments, for capturing the short term dynamics of metabolite intermediates in response to pulse experiments. To avoid the risk of blocking of the system if clump or pellet formation would occur, the sampling device was connected to a fast broth loop with an internal diameter of 8 mm. With this sampling device a constant volume of broth (1 mL) could be removed from the loop.

The circulation speed of the broth in the loop was chosen such that the travel time of the broth to the sampling device was approximately 0.25 s. It was calculated that the change in the concentration of glucose was negligible within this time interval (Fig. S6).

To test the functioning of the rapid sampling system the biomass concentrations in samples taken with the sampling device (C_x,SD_) were compared with the biomass concentrations (C_x_) in samples directly withdrawn from the fermentor (Table S4). It was observed that even though the chamber size is 1 mL, the amount of liquid sampled with the device is smaller because air is taken along. The average amount of liquid withdrawn was 0.76 (±0.04) g. Remarkably, the biomass concentration was slightly lower (6–8 %) in the samples withdrawn with the rapid sampling device. Therefore the biomass concentrations determined in these samples were used to express metabolite levels per gram dry cell weight.

#### Optimisation of cold methanol quenching fluid to avoid leakage in *A. niger*

The main purpose of this study was to develop a reliable, leakage free, quenching method for quantification of intracellular metabolites in *A. niger*, applicable to steady state chemostat cultivations as well as future dynamic pulse response experiments.

We choose the cold methanol based quenching procedure as this enables to remove extracellular metabolites, through cold centrifugation or filtration and subsequent washing with cold quenching solution. The centrifugation-based procedure for mycelia separation and washing appeared impracticable for *A. niger,* because no proper cell pellet was formed (results not shown). Therefore a quenching protocol based on cold filtration was developed, based on a previously developed procedure for *P. chrysogenum* (Douma et al. 2010).

Most importantly, the cold filtration based quenching and washing procedure, described in detail in the materials and methods section, enabled a significantly reduced contact time of the cells to the cold methanol compared to cold centrifugation, which should minimize possible leakage of metabolites. Furthermore filtration, compared to centrifugation, allows a much more efficient removal of the extracellular metabolites from the mycelium (Douma et al. 2010).

Different variations (methanol concentration and temperature) of the filtration-based cold methanol quenching fluid were applied to samples withdrawn from glucose limited chemostats carried out at dilution rates of 0.043 and 0.089 h^−1^.

Metabolite amounts were quantified in the mentioned sample fractions (intracellular, extracellular and total broth). For accurate quantification of leakage, the fraction of the washing solution should also be taken into account, however this measurement was shown to be impractical as the concentrations were too low to be quantified. Since the ^13^C labelled internal standard was added to the fractions before the extraction step in 75 % ethanol at 95 °C, possible partial degradation of metabolites was corrected for.

Quantification was carried out using LC–MS/MS and GC–MS based on isotope dilution mass spectrometry (IDMS) (Canelas et al. 2009). To enable comparison of metabolite amounts in different sample fractions, all amounts are expressed in µmol per gram biomass dry weight.

In a preliminary testing phase, 100 % aqueous methanol (v/v) solution, as used by Canelas et al. 2008 for *S. cerevisiae*, was also tested, giving low recoveries, significant leakage and low intracellular metabolite amounts (results not shown). Therefore this option was abandoned and for further optimization we zoomed in on methanol concentrations between 40 and 60 %.

**Fig. 3:**
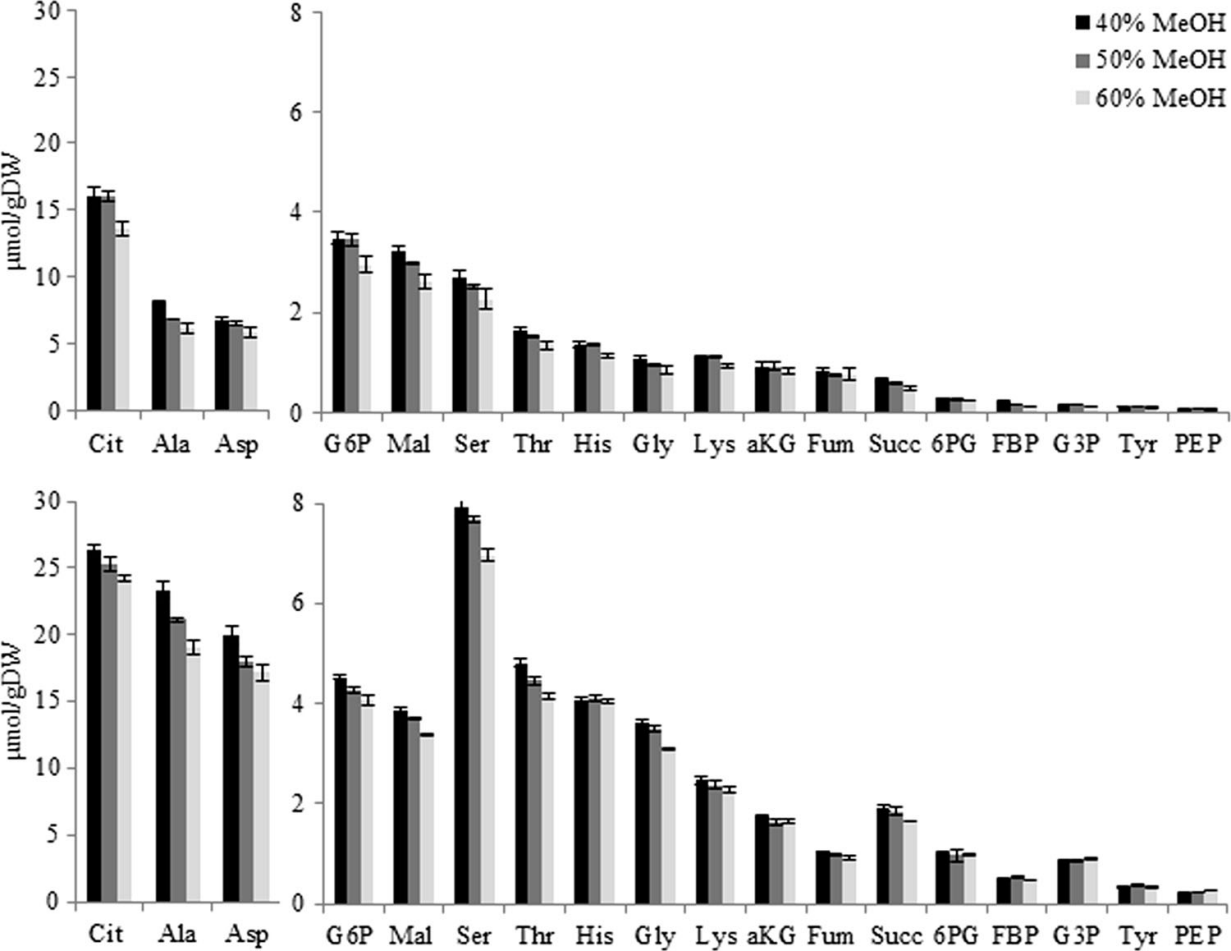
Measured intracellular metabolite amounts for different methanol concentrations of the quenching solution for *A. niger* grown at dilution rates of 0.043 h^−1^ (*upper* panel) and 0.089 h^−1^ (*lower* panel)

In Fig. 3, intracellular metabolite amounts are compared for quenching in different methanol concentrations (40, 50 and 60 %), at both dilution rates tested. Additional results can be found in the Tables S5 and S6. Clearly the intracellular metabolite level slightly dropped at 60 % methanol, compared to 40 and 50 %.

The extent of metabolite leakage brought about by the cold methanol quenching and washing of the mycelium was examined by carrying out metabolite measurements in different sample fractions (whole broth, extracellular and intracellular space). All the fractions were analysed in triplicate for this evaluation and the metabolite recoveries were calculated according to Eq. (5). The results for the two dilution rates are shown in Tables S5 and S6.

Comparing different methanol concentrations, the average metabolite recovery and respective standard error was 79 % (±10 %) for 40 % aqueous methanol (v/v), 77 % (±9 %) for 50 % aqueous methanol (v/v), and 67 % (±9 %) for 60 % aqueous methanol (v/v) for 0.043 h^−1^ dilution rate. For the higher dilution rate (0.089 h^−1^), the recoveries were: 95 % (±4 %) for 40 % aqueous methanol (v/v), 93 % (±5 %) for 50 % aqueous methanol (v/v), and 89 % (±4 %) for 60 % aqueous methanol (v/v). Thus, for both dilution rates the recoveries were highest for the 40 % aqueous methanol solution.

These results show that quenching at −20 °C in 40 % aqueous methanol (v/v) appears to be the best quenching procedure for *A. niger* intracellular metabolomics. From previous studies it can be inferred that the optimal cold methanol quenching conditions are quite different for different eukaryotic microorganisms, as it has been found that for *S. cerevisiae, P. pastoris and P. chrysogenum* leakage was minimal for methanol concentrations of 100 % (−40 °C), 60 % (−27 °C) and 40 % (−20 °C) respectively (Canelas et al. 2008; Carnicer et al. 2012; de Jonge et al. 2012). Remarkably, the optimal quenching condition found for *A. niger* in this study is the same as has been reported for *P. chrysogenum*.

Comparing the metabolite levels of the different dilution rates it is clear that the higher dilution rate leads to higher metabolite amounts, which is to be expected due to higher fluxes. Similar results were found in *S. cerevisiae* (Canelas et al. 2009).

**Fig. 4:**
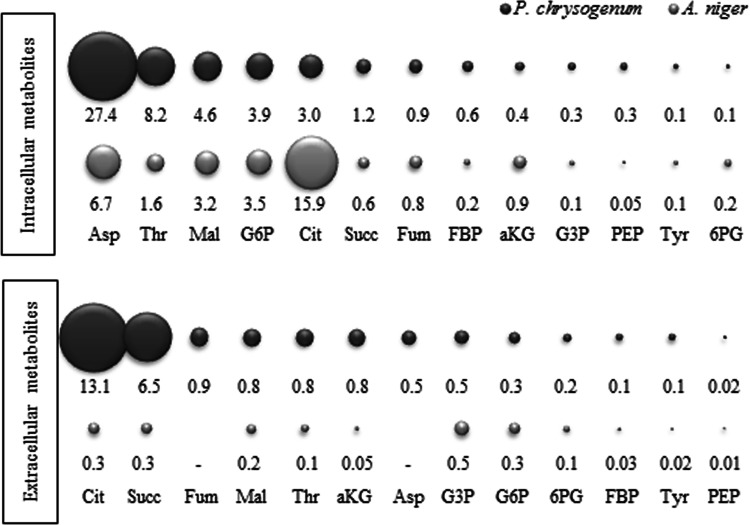
Intracellular and extracellular metabolite comparison of *A. niger* and *P. chrysogenum* at a comparable dilution rate (0.043 and 0.05 h^−1^). The bubble areas are proportional to the pool sizes of the metabolites in µmol/g_DW_

The levels of intra- and extracellular metabolites determined for *A. niger* grown in an aerobic glucose limited chemostat at a dilution rate of 0.043 h^−1^, were compared with the levels reported for *P. chrysogenum* grown under similar chemostat conditions (D = 0.05 h^−1^), but at a significantly higher pH 6.5 (de Jonge et al. 2012) (Fig. 4). For *A. niger* the measured metabolite levels were between 0.02 (GAP) and 67 µmol/g DW (Tre) a difference of 4 orders of magnitude (Table S6). Similarly, the published values for *P. chrysogenum* also vary 4 orders of magnitude between 0.09 (Met) and 12 µmol/g DW (Cit).

Nevertheless, intracellular metabolite pools of both *A. niger* and *P. chrysogenum* are comparable. This is to be expected because, as both organisms were cultured at similar growth rates in glucose limited chemostats, the fluxes in central metabolism in both systems should be very similar. Remarkable differences between both fungi are the intracellular levels of citrate, of which the level is five times higher in *A. niger* and threonine and aspartic acid, which are five and six times higher in *P. chrysogenum*. Citric acid is a known product in *A. niger* species as mentioned before, and threonine was found in other Penicillium studies, in the same range of intracellular amount here compared (Nasution et al. 2006).

For both organisms the extracellular metabolite amounts are considerably lower than the intracellular ones, with exception of citric acid and succinic acid which were both present in considerable amounts in the extracellular space of *P. chrysogenum*.

## Concluding remarks

The purpose of this work was to develop a chemostat protocol for homogeneous steady state cultivation of *A. niger* in a conventional turbine stirred bioreactor, to develop and validate a robust rapid sampling device for filamentous fungi, and to perform a systematic investigation of cold methanol quenching combined with cold filtration to enable quantitative metabolomics studies of this organism.

We demonstrated that using the developed chemostat protocol, *A. niger* is able to grow as homogeneous, finely dispersed mycelium in steady state glucose limited chemostat cultures without significant wall growth. Furthermore we developed a rapid sampling device and an efficient cold methanol quenching method combined with rapid cold filtration for rapid sampling, harvesting and washing of the mycelium enabling accurate quantitative metabolomics studies. A 40 % (v/v) aqueous methanol mixture at −20 °C resulted in minimal metabolite leakage. The application of our validated techniques provided consistent data. A comparison with published metabolome data of *P. chrysogenum*, grown in similar chemostats revealed similar metabolome profiles and some interesting differences. Overall, with this work we now have validated techniques for metabolomics studies of *A. niger*, as a starting point for further metabolomics and metabolite transport studies.

## Electronic supplementary material

Below is the link to the electronic supplementary material.

Supplementary material 1 (PPTX 2,330 kb)

Supplementary material 2 (DOCX 31 kb)

## Abbreviations

$\gamma_{x}$: Degree of reduction of compound x
µ: Growth rate (h^−1^)
aa: Amino acid
C_x_: Biomass concentration inside the reactor
C_x,out_: Biomass concentration in the outflow of the reactor
g_DW_: Grams of dry cell weight
Gly: Glycolytic metabolites
MeOH: Methanol
PPP: Pentose phosphate pathway
R_x_: Net conversion rate of compound x (Cmol/h)
TCA: Tricarboxylic acid cycle
TOC: Total organic carbon

## References

[CR1] Canelas AB (2008). Leakage-free rapid quenching technique for yeast metabolomics. Metabolomics.

[CR2] Canelas AB (2009). Quantitative evaluation of intracellular metabolite extraction techniques for yeast metabolomics. Analytical Chemistry.

[CR3] Carnicer M (2012). Development of quantitative metabolomics for *Pichia pastoris*. Metabolomics.

[CR4] Cipollina C (2009). A comprehensive method for the quantification of the non-oxidative pentose phosphate pathway intermediates in *Saccharomyces cerevisiae* by GC-IDMS. Journal of Chromatography, B: Analytical Technologies in the Biomedical and Life Sciences.

[CR5] De Jonge LP (2011). Scale-down of penicillin production in *Penicillium chrysogenum*. Biotechnology Journal.

[CR6] De Jonge LP (2012). Optimization of cold methanol quenching for quantitative metabolomics of *Penicillium chrysogenum*. Metabolomics.

[CR7] De Koning W, van Dam K (1992). A method for the determination of changes of glycolytic metabolites in yeast on a subsecond time scale using extraction at neutral pH. Analytical Biochemistry.

[CR8] Douma RD (2010). Intracellular metabolite determination in the presence of extracellular abundance: Application to the penicillin biosynthesis pathway in *Penicillium chrysogenum*. Biotechnology and Bioengineering.

[CR9] Jernejc K (2004). Comparison of different methods for metabolite extraction from *Aspergillus niger* mycelium. Acta Chimica Slovenica.

[CR10] Jørgensen TR (2007). Glucose uptake and growth of glucose-limited chemostat cultures of Aspergillus niger and a disruptant lacking MstA, a high-affinity glucose transporter. Microbiology (Reading, England).

[CR11] Jørgensen TR (2011). Submerged conidiation and product formation by *Aspergillus niger* at low specific growth rates are affected in aerial developmental mutants. Applied and Environmental Microbiology.

[CR12] Lange HC (2001). Improved rapid sampling for in vivo kinetics of intracellular metabolites in *Saccharomyces cerevisiae*. Biotechnology and Bioengineering.

[CR13] Larsen B (2004). Homogeneous batch cultures of *Aspergillus oryzae* by elimination of wall growth in the Variomixing bioreactor. Applied Microbiology and Biotechnology.

[CR14] Larsson G, Törnkvist M (1996). Rapid sampling, cell inactivation and evaluation of low extracellular glucose concentrations during fed-batch cultivation. Journal of Biotechnology.

[CR15] Mashego MR (2003). Critical evaluation of sampling techniques for residual glucose determination in carbon-limited chemostat culture of *Saccharomyces cerevisiae*. Biotechnology and Bioengineering.

[CR16] Mashego MR (2004). MIRACLE: Mass isotopomer ratio analysis of U-13C-labeled extracts. A new method for accurate quantification of changes in concentrations of intracellular metabolites. Biotechnology and Bioengineering.

[CR17] Nasution U (2006). Measurement of intracellular metabolites of primary metabolism and adenine nucleotides in chemostat cultivated *Penicillium chrysogenum*. Biotechnology and Bioengineering.

[CR18] Pedersen H, Beyer M, Nielsen J (2000). Glucoamylase production in batch, chemostat and fed-batch cultivations by an industrial strain of *Aspergillus niger*. Applied Microbiology and Biotechnology.

[CR19] Pel HJ (2007). Genome sequencing and analysis of the versatile cell factory *Aspergillus niger* CBS 513.88. Nature Biotechnology.

[CR20] Roels JA (1983). Energetics and kinetics in biotechnology.

[CR21] Ruijter GJG, Visser J (1996). Determination of intermediary metabolites in *Aspergillus niger*. Journal of Microbiological Methods.

[CR22] Schädel F, Franco-Lara E (2009). Rapid sampling devices for metabolic engineering applications. Applied Microbiology and Biotechnology.

[CR23] Schrickx JM (1993). Growth and product formation in chemostat and recycling cultures by *Aspergillus niger* N402 and a glucoamylase overproducing transformant, provided with multiple copies of the glaA gene. Journal of General Microbiology.

[CR24] Swift RJ (1998). Recombinant glucoamylase production by *Aspergillus niger* B1 in chemostat and pH auxostat cultures. Fungal Genetics and Biology: FG and B.

[CR25] Taymaz-Nikerel H (2009). Development and application of a differential method for reliable metabolome analysis in *Escherichia coli*. Analytical Biochemistry.

[CR26] Van Dam JC (2002). Analysis of glycolytic intermediates in *Saccharomyces cerevisiae* using anion exchange chromatography and electrospray ionization with tandem mass spectrometric detection. Analytica Chimica Acta.

[CR27] Van Gulik WM (2000). Application of metabolic flux analysis for the identification of metabolic bottlenecks in the biosynthesis of penicillin-G. Biotechnology and Bioengineering.

[CR28] Verheijen PJT, Smolke C (2010). Data reconciliation and error detection. balances and reaction models. The metabolic pathway engineering handbook.

[CR29] Vishniac W, Santer M (1957). The thiobacilli. Bacteriological Reviews.

[CR30] Werpy, T., et al. (2004). Top value added chemicals from biomass. Volume 1-results of screening for potential candidates from sugars and synthesis gas. *U.S. Department of Energy*. Available at: http://www.nrel.gov/docs/fy04osti/35523.pdf.

[CR31] Wu L (2005). Quantitative analysis of the microbial metabolome by isotope dilution mass spectrometry using uniformly ^13^C-labeled cell extracts as internal standards. Analytical Biochemistry.

