# Supplementary material for: Development of tools for quantitative intracellular metabolomics of Aspergillus niger chemostat cultures
Source: Metabolomics. 2015 Feb 25;11(5):1253–64. doi: 10.1007/s11306-015-0781-z (PMC4559092; doi:10.1007/s11306-015-0781-z)
Supplement: Supplementary file 1 — Supplementary material 1 (PPTX 2,330 kb) [file 11306_2015_781_MOESM1_ESM.pptx]

## Slide 1
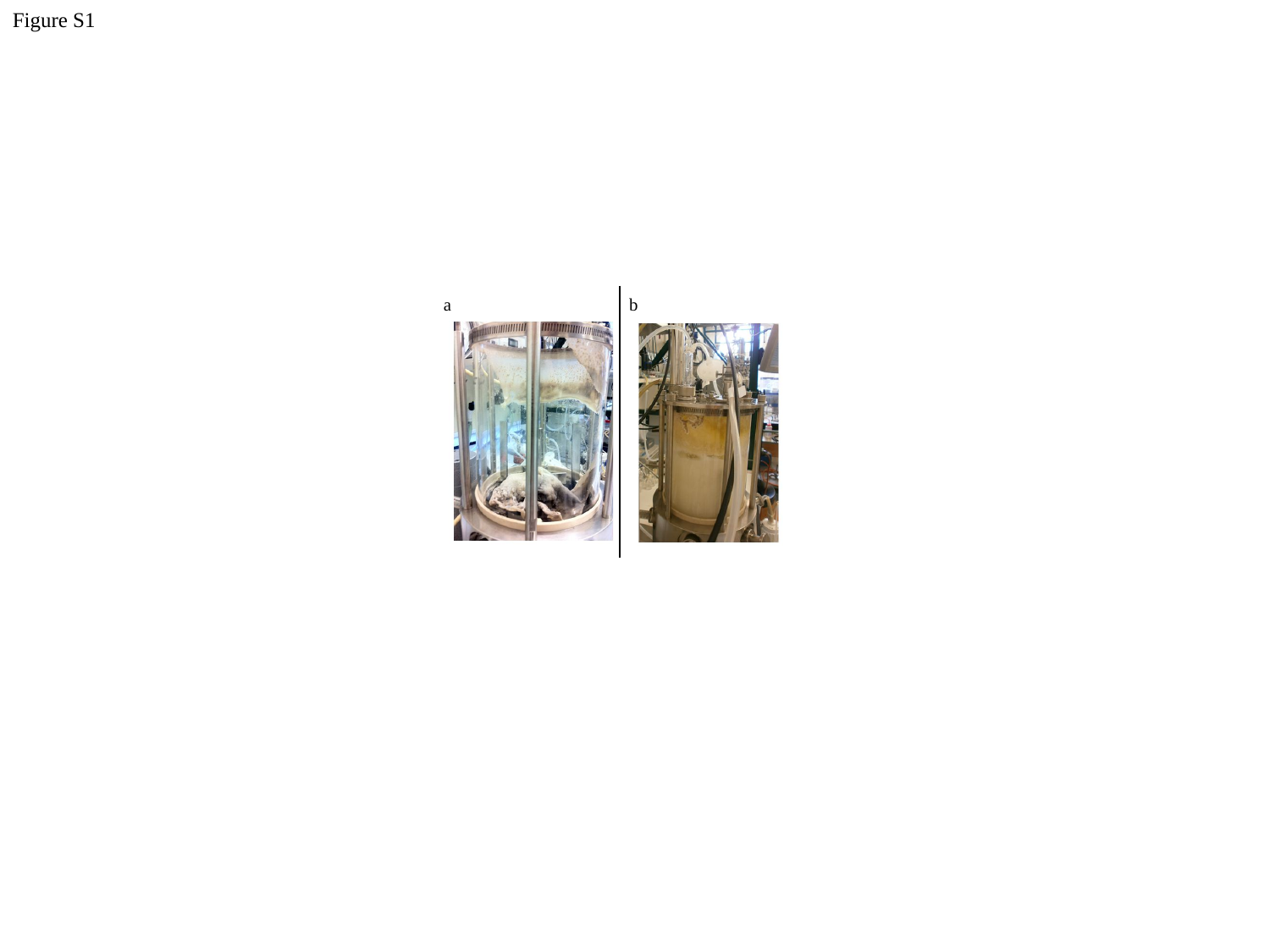

Figure S1
| a | b |
| --- | --- |

## Slide 2
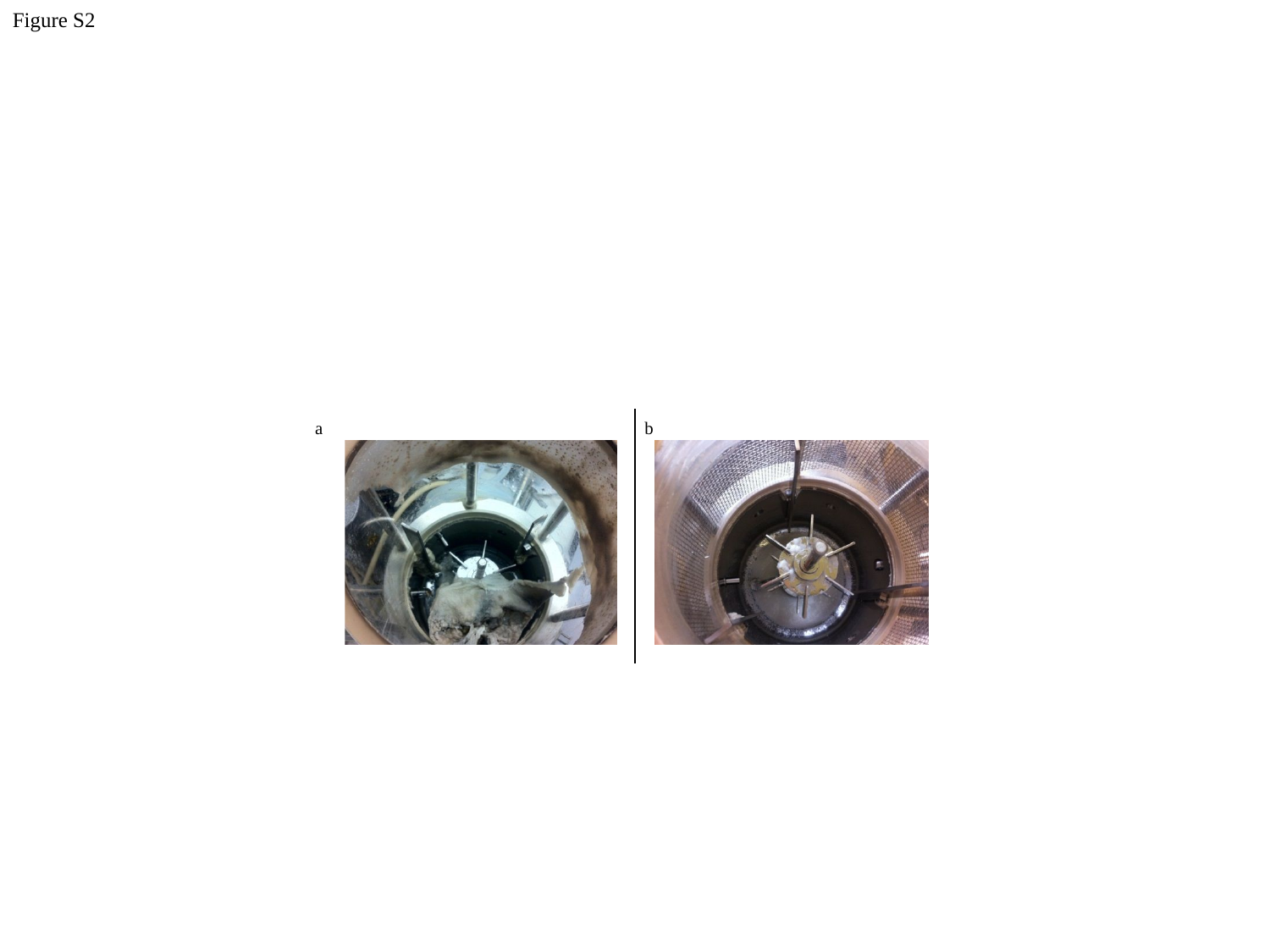

Figure S2
| a | b |
| --- | --- |

## Slide 3
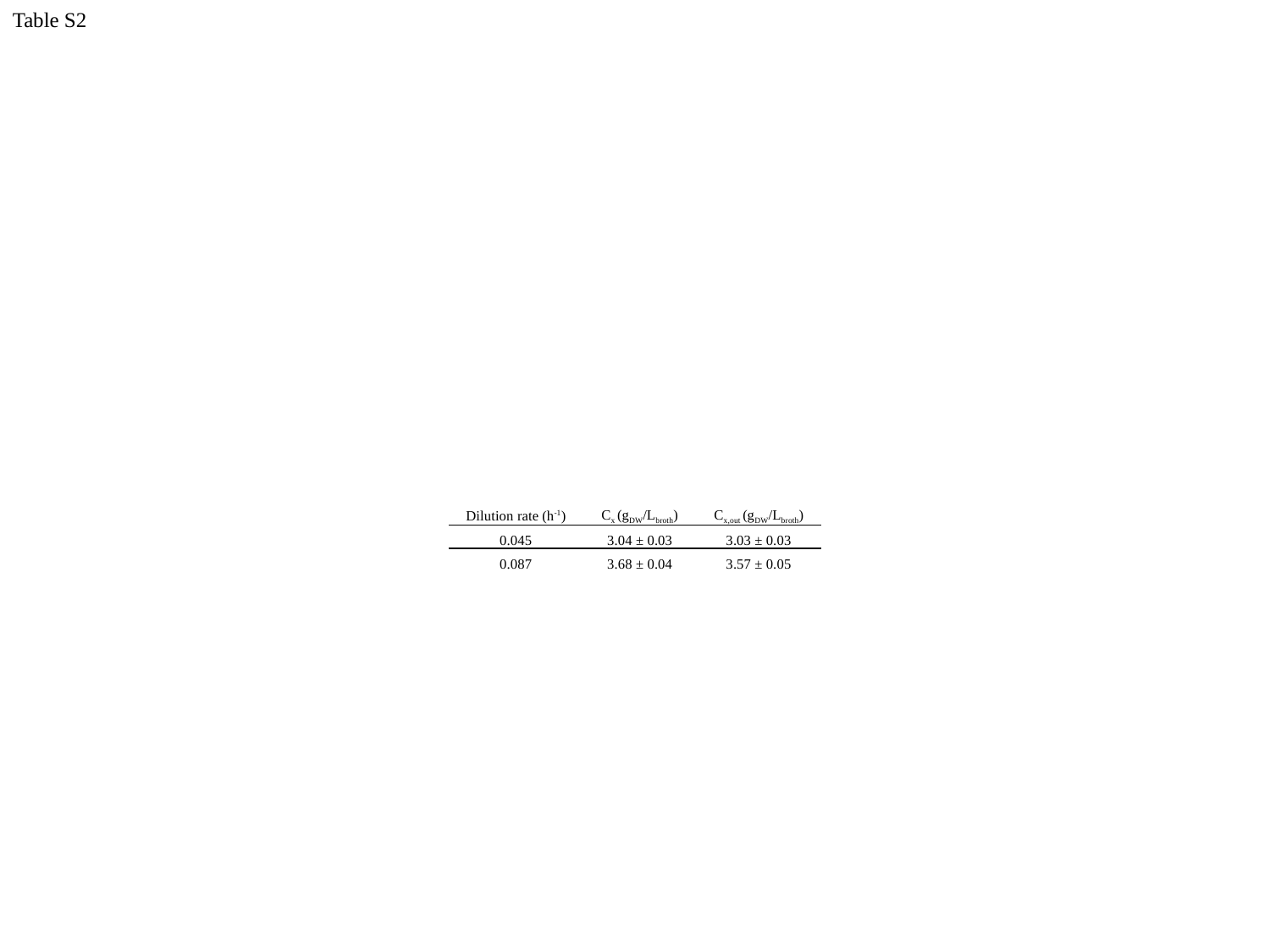

Table S2
| Dilution rate (h-1) | Cx (gDW/Lbroth) | Cx,out (gDW/Lbroth) |
| --- | --- | --- |
| 0.045 | 3.04 ± 0.03 | 3.03 ± 0.03 |
| 0.087 | 3.68 ± 0.04 | 3.57 ± 0.05 |

## Slide 4
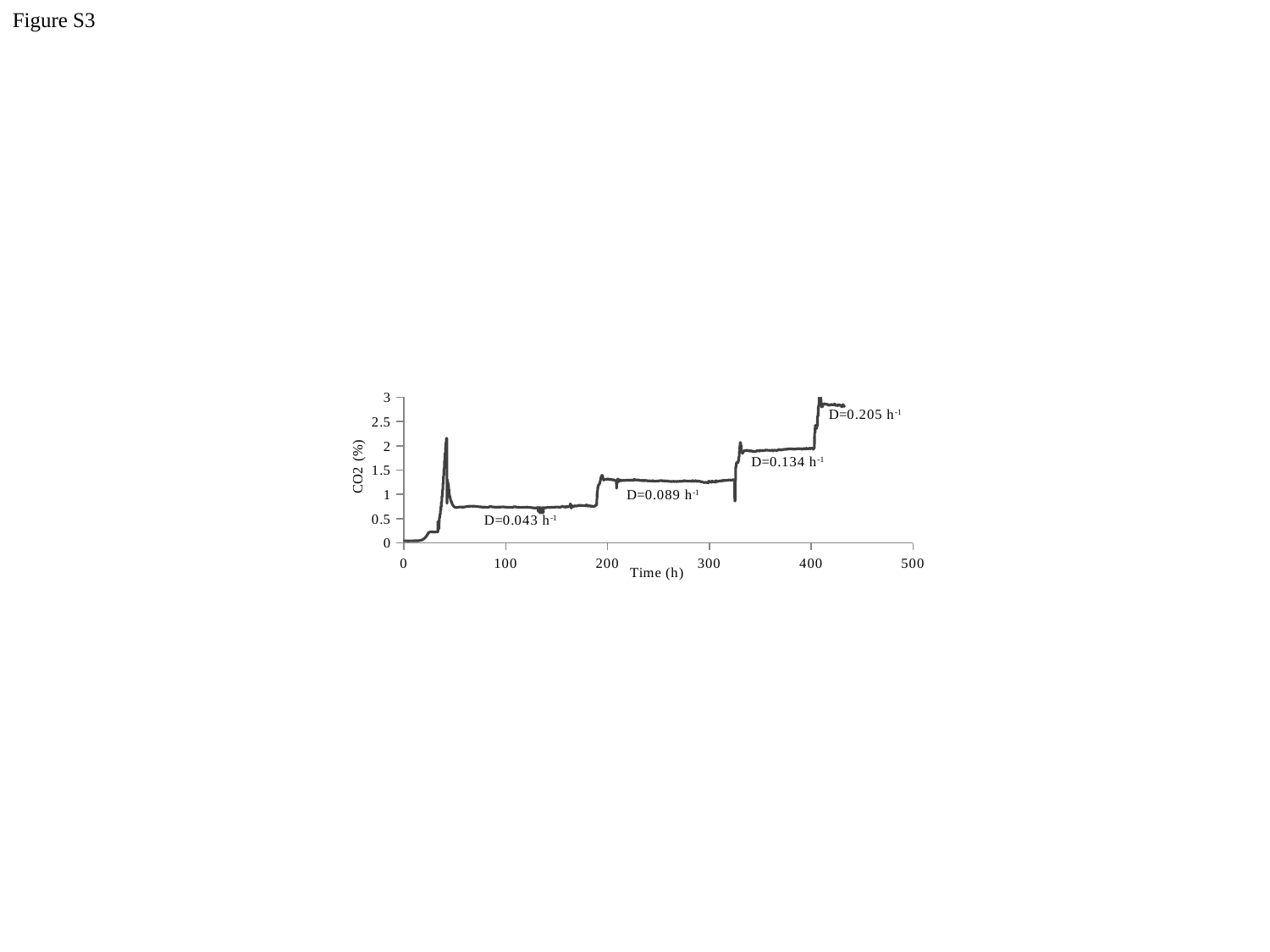

Figure S3
### Chart
| Category | Offgas_CO2_Value |
|---|---|

## Slide 5
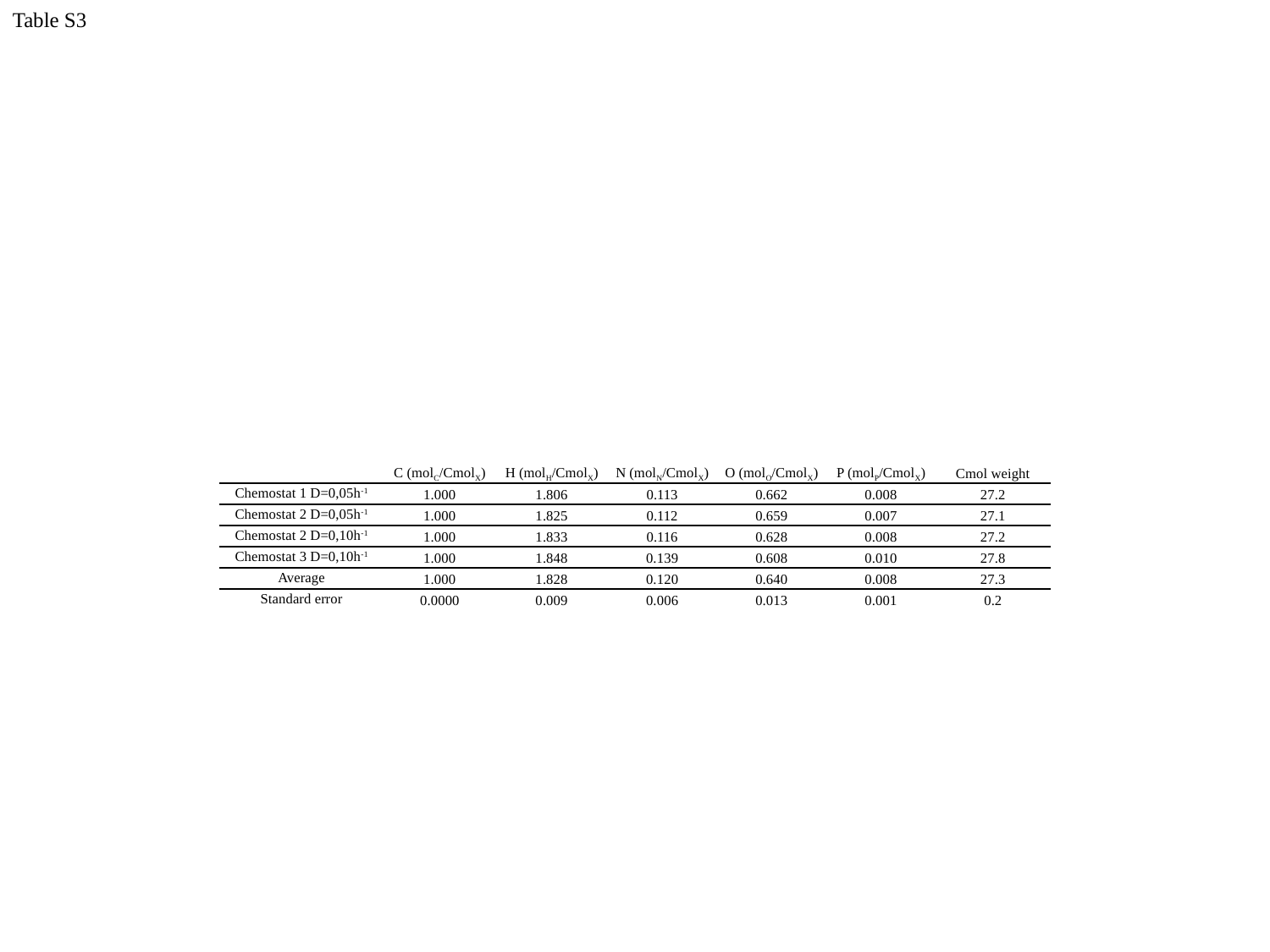

Table S3
| | C (molC/CmolX) | H (molH/CmolX) | N (molN/CmolX) | O (molO/CmolX) | P (molP/CmolX) | Cmol weight |
| --- | --- | --- | --- | --- | --- | --- |
| Chemostat 1 D=0,05h-1 | 1.000 | 1.806 | 0.113 | 0.662 | 0.008 | 27.2 |
| Chemostat 2 D=0,05h-1 | 1.000 | 1.825 | 0.112 | 0.659 | 0.007 | 27.1 |
| Chemostat 2 D=0,10h-1 | 1.000 | 1.833 | 0.116 | 0.628 | 0.008 | 27.2 |
| Chemostat 3 D=0,10h-1 | 1.000 | 1.848 | 0.139 | 0.608 | 0.010 | 27.8 |
| Average | 1.000 | 1.828 | 0.120 | 0.640 | 0.008 | 27.3 |
| Standard error | 0.0000 | 0.009 | 0.006 | 0.013 | 0.001 | 0.2 |

## Slide 6
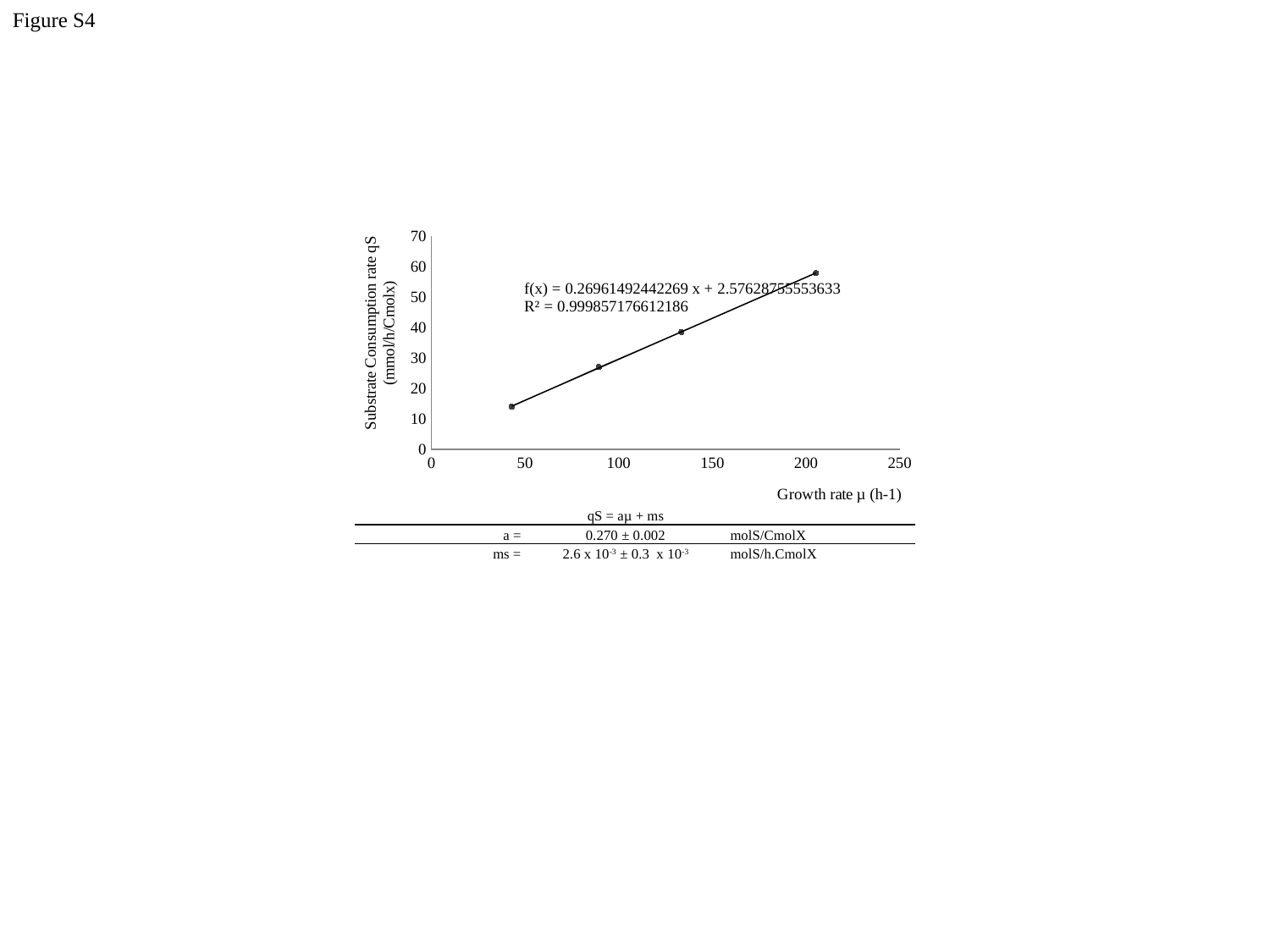

Figure S4
### Chart
| Category | |
|---|---|
| | qS = aµ + ms | |
| --- | --- | --- |
| a = | 0.270 ± 0.002 | molS/CmolX |
| ms = | 2.6 x 10^-3^ ± 0.3 x 10^-3^ | molS/h.CmolX |

## Slide 7
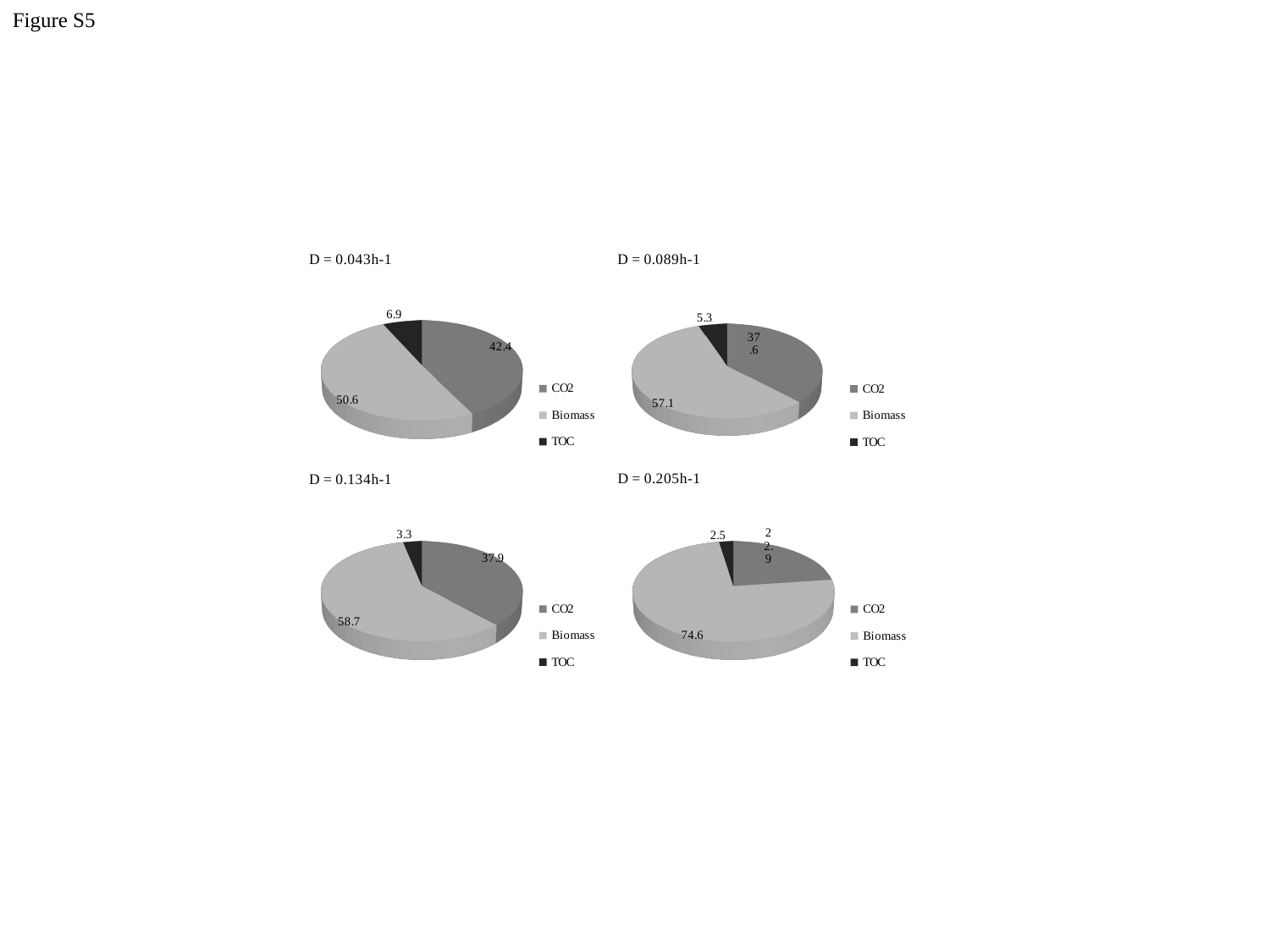

Figure S5
[unsupported chart]
[unsupported chart]
[unsupported chart]
[unsupported chart]

## Slide 8
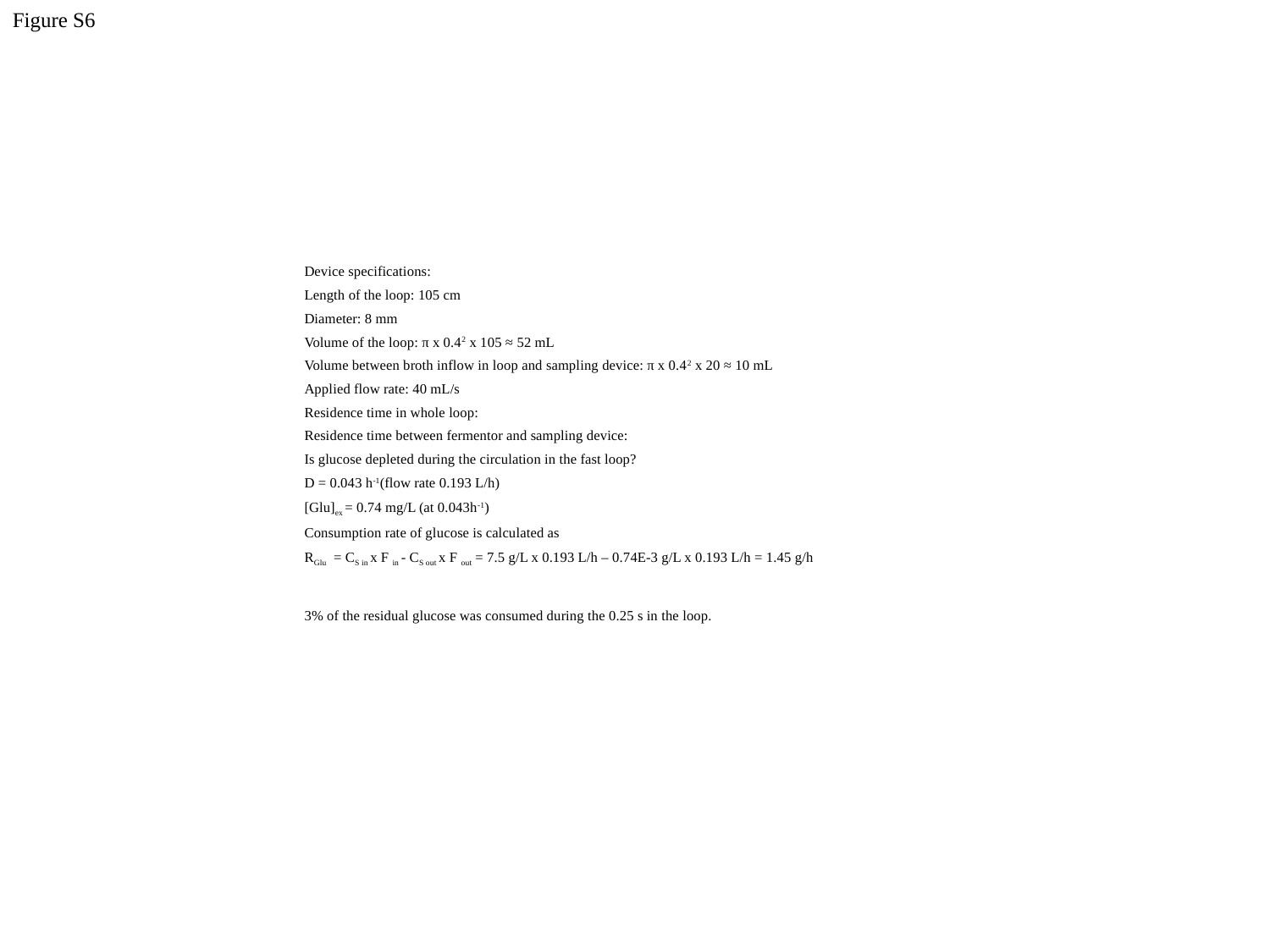

Figure S6

## Slide 9
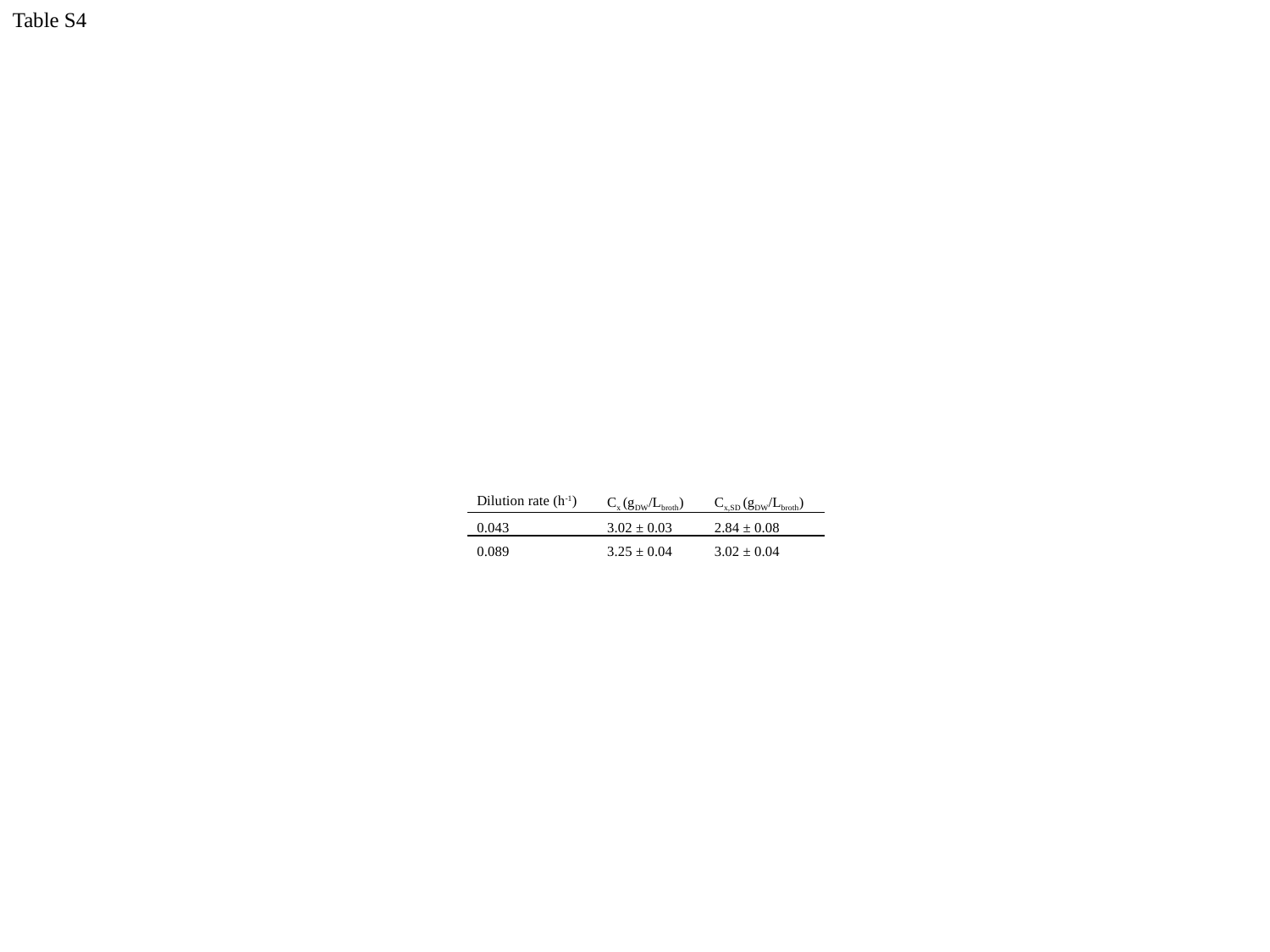

Table S4
| Dilution rate (h-1) | Cx (gDW/Lbroth) | Cx,SD (gDW/Lbroth) |
| --- | --- | --- |
| 0.043 | 3.02 ± 0.03 | 2.84 ± 0.08 |
| 0.089 | 3.25 ± 0.04 | 3.02 ± 0.04 |

## Slide 10
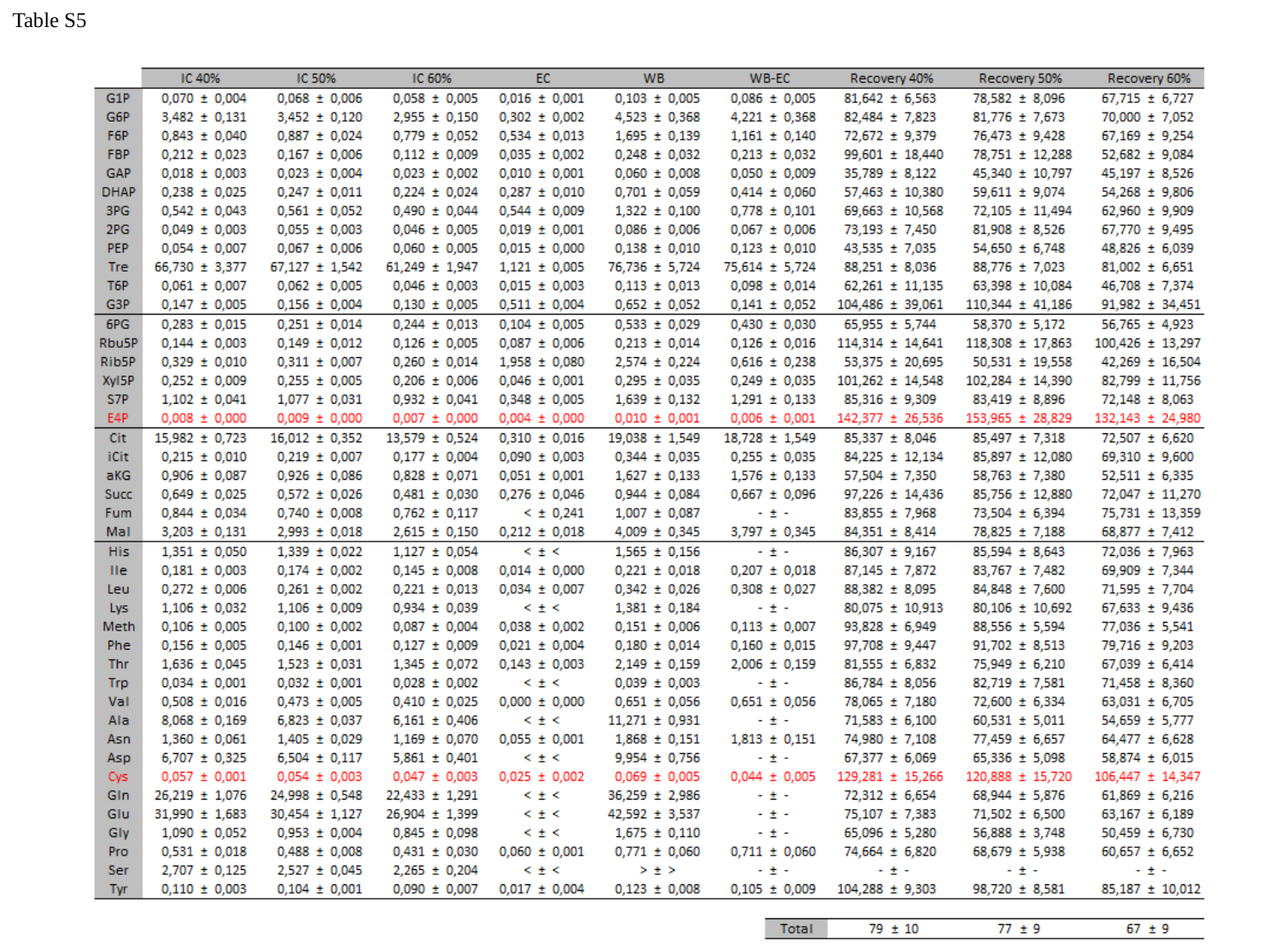

Table S5

## Slide 11
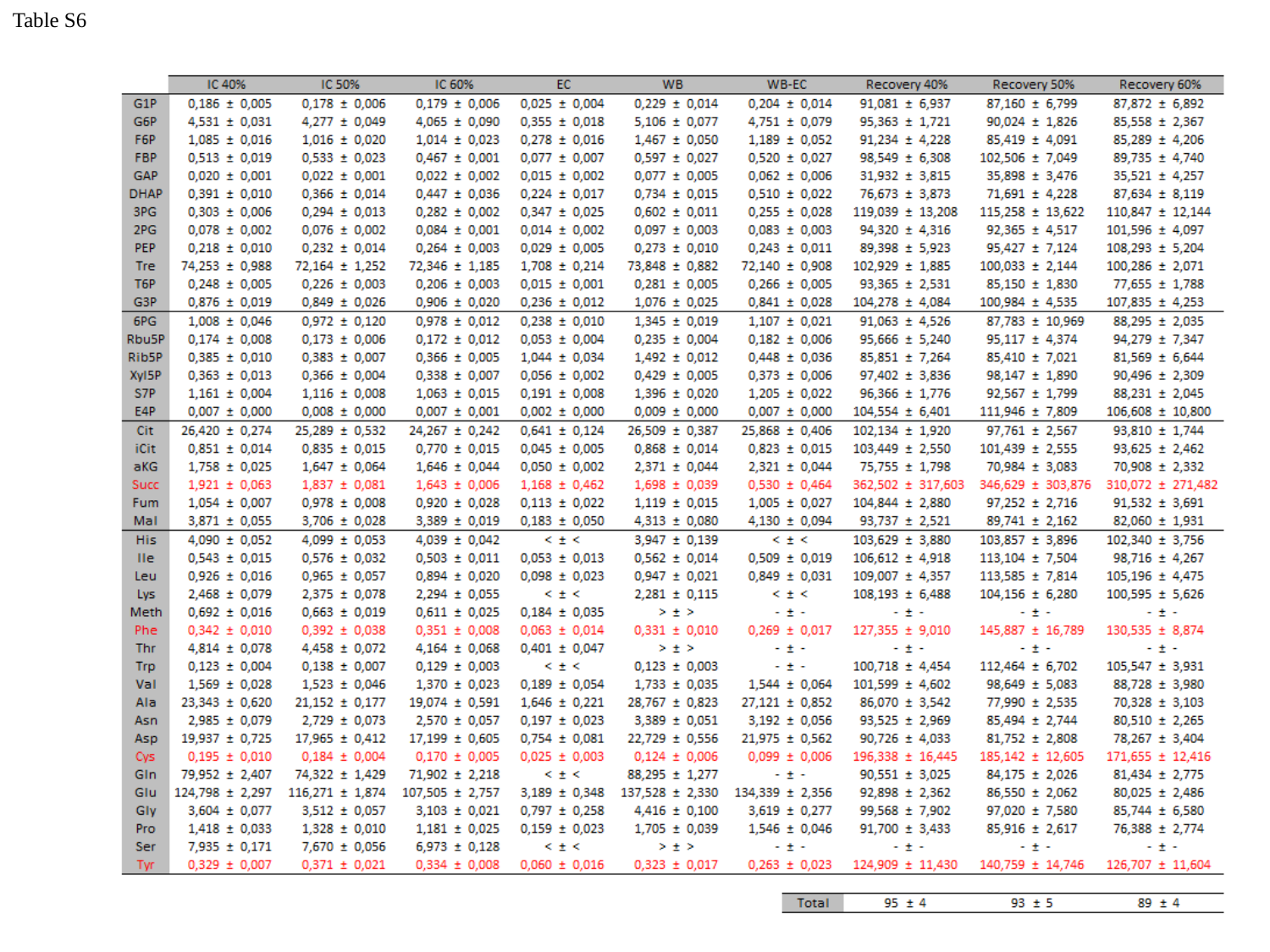

Table S6
