# Supplementary material for: Development of tools for quantitative intracellular metabolomics of Aspergillus niger chemostat cultures
Source: Metabolomics. 2015 Feb 25;11(5):1253–64. doi: 10.1007/s11306-015-0781-z (PMC4559092; doi:10.1007/s11306-015-0781-z)
Supplement: Supplementary file 2 — Supplementary material 2 (DOCX 31 kb) [file 11306_2015_781_MOESM2_ESM.docx]

**Development of tools for quantitative intracellular metabolomics of *Aspergillus niger* chemostat cultures**

**Quantitative metabolomics of *A. niger* chemostat cultures** (abbreviated tittle)

Francisca Lameiras, Joseph J Heijnen, Walter M van Gulik^*^

Cell Systems Engineering section, Department of Biotechnology, Delft University of Technology, 2628 BC Delft, Netherlands

^*^Corresponding author

W.M.vangulik@tudelft.nl, T. +31 15 278 4629

**Supplementary material**

**Table S1.** Parameters of the 43 metabolites identified in the investigated samples.

|  | **Metabolite** | **Abbr.** | **PubChem CID** | **Method** | **Derivate** | **Quantification ion ^12^C (precursor/product) [*m/z*]** | **Quantification ion internal standard ^13^C (precursor/product) [*m/z*]** |
| --- | --- | --- | --- | --- | --- | --- | --- |
| 1 | 2-phosphoglycerate | 2PG | 59 | Cipollina et al. 2009 | 4TMS | 459.1 | 462.1 |
| 2 | 3-phosphoglycerate | 3PG | 724 | Cipollina et al. 2009 | 4TMS | 459.1 | 462.1 |
| 3 | 6-phosphogluconate | 6PG | 91493 | Van Dam *et al.* 2002 | n.a. | 275/96.7 | 281/96.7 |
| 4 | Ketoglutarate | aKG | 51 | Cipollina et al. 2009 | 2TMS 1MOX | 304.0 | 309.0 |
| 5 | Alanine | Ala | 5950 | de Jonge *et al.* 2011 | 2TBDMS | 260.1 | 263.1 |
| 6 | Asparagine | Asn | 6267 | de Jonge *et al.* 2011 | 3TBDMS | 417.2 | 421.2 |
| 7 | Aspartic acid | Asp | 424 | de Jonge *et al.* 2011 | 3TBDMS | 418.2 | 422.2 |
| 8 | Citrate | Cit | 311 | Cipollina et al. 2009 | 4TMS | 465.1 | 471.1 |
| 9 | Cysteine | Cys | 5862 | de Jonge *et al.* 2011 | 3TBDMS | 406.1 | 409.1 |
| 10 | Dihydroxyacetone phosphate | DHAP | 668 | Cipollina et al. 2009 | 3TMS 1MOX | 400.1 | 403.1 |
| 11 | Erythrose-4-phosphate | E4P | 122357 | Cipollina et al. 2009 | 4TMS 1MOX | 357.1 | 359.1 |
| 12 | Fructose-6-phosphate | F6P | 69507 | Cipollina et al. 2009 | 6TMS 1MOX | 459.1 | 462.1 |
| 13 | Fructose-1,6-bis phosphate | FBP | 10267 | Van Dam *et al.* 2002 | n.a. | 339/96.7 | 345/96.7 |
| 14 | Fumaric acid | Fum | 44972 | Cipollina et al. 2009 | 2TMS | 245.0 | 249.0 |
| 15 | Glycerol-3-phosphate | G3P | 754 | Van Dam *et al.* 2002 | n.a. | 171/78.8 | 174/78.8 |
| 16 | Glucose-6-phosphate | G6P | 5958 | Cipollina et al. 2009 | 6TMS 2MOX | 471.0 | 475.0 |
| 17 | Glyceraldehyde-3-phosphate | GAP | 729 | Cipollina et al. 2009 | 3TMS 1MOX | 400.1 | 403.1 |
| 18 | Glucose | Glc | 79025 | Cipollina et al. 2009 | 5TMS 1MOX | 319.1 | 323.1 |
| 19 | Glutamine | Gln | 5961 | de Jonge *et al.* 2011 | 3TBDMS | 431.2 | 436.2 |
| 20 | Glutamic acid | Glu | 611 | de Jonge *et al.* 2011 | 3TBDMS | 474.3 | 479.4 |
| 21 | Glycine | Gly | 750 | de Jonge *et al.* 2011 | 2TBDMS | 246.1 | 148.1 |
| 22 | Histidine | His | 6274 | de Jonge *et al.* 2011 | 3TBDMS | 440.3 | 446.3 |
| 23 | isocitrate | iCit | 1198 | Cipollina et al. 2009 | 4TMS | 465.1 | 471.1 |
| 24 | isoleucine | Ile | 6306 | de Jonge *et al.* 2011 | 2TBDMS | 274.1 | 279.1 |
| 25 | Leucine | Leu | 74840 | de Jonge *et al.* 2011 | 2TBDMS | 274.1 | 279.1 |
| 26 | Lysine | Lys | 5962 | de Jonge *et al.* 2011 | 3TBDMS | 431.3 | 437.1 |
| 27 | Malate | Mal | 525 | Cipollina et al. 2009 | 3TMS | 335.0 | 339.0 |
| 28 | Methionine | Meth | 6137 | de Jonge *et al.* 2011 | 2TBDMS | 320.1 | 325.1 |
| 29 | Phosphoenolpyruvate | PEP | 1005 | Van Dam *et al.* 2002 | n.a. | 167/78.8 | 170/78.8 |
| 30 | Phenylalanine | Phe | 6140 | de Jonge *et al.* 2011 | 2 TBDMS | 336.2 | 345.2 |
| 31 | Proline | Pro | 145742 | de Jonge *et al.* 2011 | 2 TBDMS | 286.1 | 291.1 |
| 32 | Ribose-5-phosphate | Rib5P | 21115541 | Cipollina et al. 2009 | 5TMS 1MOX | 459.1 | 462.1 |
| 33 | Ribulose-5-phosphate | Ribu5P | 439184 | Cipollina et al. 2009 | 5TMS 1MOX | 357.1 | 359.1 |
| 34 | Sedoheptulose-7-phosphate | S7P | 165007 | Cipollina et al. 2009 | 7TMS 1MOX | 471.1 | 475.1 |
| 35 | Serine | Ser | 5951 | de Jonge *et al.* 2011 | 3 TBDMS | 390.2 | 393.2 |
| 36 | Succinate | Succ | 160419 | Van Dam *et al.* 2002 | n.a. | 117/72.7 | 121/75.7 |
| 37 | Trehalose6-phosphate | T6P | 122336 | Van Dam *et al.* 2002 | n.a. | 421.2/78.8 | 433.2/78.8 |
| 38 | Threonine | Thr | 6288 | de Jonge *et al.* 2011 | 3TBDMS | 404.2 | 408.2 |
| 39 | Trehalose | Tre | 7427 | Cipollina et al. 2009 | 8TMS | 361.1 | 367.1 |
| 40 | Tryptophan | Trp | 6305 | de Jonge *et al.* 2011 | 2TBDMS | 375.2 | 386.2 |
| 41 | Tyrosine | Tyr | 6057 | de Jonge *et al.* 2011 | 3TBDMS | 466.3 | 475.3 |
| 42 | Valine | Val | 6287 | de Jonge *et al.* 2011 | 2TBDMS | 288.1 | 293.1 |
| 43 | Xylulose-5-phosphate | Xyl5P | 5459820 | Cipollina et al. 2009 | 5TMS 1MOX | 357.0 | 359.0 |

Figure S1. *A. niger* wall growth accumulation in the fermentor during fermentation (a) and before cleaning (b).

Figure S2. Top view of the fermentor. Massive aggregation of biomass with a conventional fermentor set up (a) in comparison to a biofilm-free bioreactor after optimization of the fermentor setup (b).

Table S2. Biomass concentration.

Figure S3. CO_2_ profile during chemostat phase: consecutive steady states.

Table S3. Average biomass elemental composition of *A. niger* (calculation based on three different chemostats).

**Figure S4.** Herbert Pirt relation.

**Figure S5.** Distribution of carbon supplied as substrate (glucose).

**Figure S6.** Example calculation of glucose depletion in the broth loop**.**

Table S4. Biomass concentration inside the fermentor C_x_ and in samples from the sampling device C_x,SD._

**Table S5.** Measured metabolite amounts in different sample fractions (µmol/g_DW_) at dilution rate 0.043 h^-1^. Averages and standard errors of triplicate samples are shown.

In (red) are measurements which were judged to be inconsistent due to concentrations being close to the detection limit, and in (<), measurements below the detection limit.

**Table S6.** Measured metabolite amounts in different sample fractions (µmol/g_DW_) at dilution rate 0.089 h^-1^. Averages and standard errors of triplicate samples are shown.

In (red) are measurements which were judged to be inconsistent due to concentrations being close to the detection limit, and in (<), measurements below the detection limit.
